# Using implementation research frameworks to identify facilitators and barriers to implementation of ferric carboxymaltose intravenous iron among moderately anemic postpartum women participating in a trial in India

**DOI:** 10.1371/journal.pone.0359787

**Published:** 2026-10-21

**Authors:** Umesh Charantimath, Valerie L. Flax, Ashwini Dandappanavar, Anika Hannan, Mrityunjay C. Metgud, Avinash Kavi, Manjunath Somannavar, Shivaprasad S. Goudar, Simal Thind, Rosemary Frasso, Lauren Wiener, Sabrina LaRosa, Cecilia Rasco, Elizabeth M. McClure, Richard Derman

**Affiliations:** 1 Women’s and Children’s Health Research Unit, Jawaharlal Nehru Medical College, KLE Academy of Higher Education and Research, Belagavi, Karnataka, India; 2 RTI International, Durham, NC, United States of America; 3 Jefferson College of Population Health, Thomas Jefferson University, Philadelphia, Pennsylvania, United States of America; 4 Asano-Gonnella Center for Research in Medical Education and Health Care, Sidney Kimmel Medical College, Thomas Jefferson University, Philadelphia, Pennsylvania, United States of America; 5 Department of Global Affairs, Thomas Jefferson University, Philadelphia, Pennsylvania, United States of America; Nelson Mandela African Institute of Science and Technology, TANZANIA, UNITED REPUBLIC OF

## Abstract

Postpartum anemia affects approximately 75% of women in India. We conducted this implementation research (IR) related to the use of ferric carboxymaltose (FCM) intravenous (IV) iron as part of a trial in anemic postpartum women. The aim of the IR study was to understand potential facilitators and barriers to implementation of single-dose FCM IV iron. This is a mixed-methods study in three secondary-level healthcare facilities in Belagavi, India, designed to explore selected Consolidated Framework for Implementation Research constructs and Proctor’s framework elements. Quantitative data were gathered via surveys of postpartum women who received IV FCM (n = 308) and from healthcare workers and administrators (n = 16). Qualitative data included in-depth interviews with 15 women who received IV FCM, 15 family members of women, and 15 healthcare workers and administrators. Quantitative data were analyzed descriptively, and interview transcripts underwent directed content analysis to augment survey findings. Nearly all women believed IV iron made anemia symptoms better, 76% felt the single infusion process was very easy, 58% completely agreed to receive treatment again, and 69% were very likely to recommend treatment to family or friends. Family members were supportive of women receiving FCM. Health workers felt that single dose FCM had comparative advantages over oral iron; however, 4/10 health workers said giving FCM IV iron is harder than oral iron because of the patient monitoring involved and 8/16 health workers and administrators felt there were not enough trained staff to provide the service. Fewer than half of health workers and administrators (7/16) believed their hospital had enough resources to implement IV FCM infusions, and 9/16 said getting the necessary supplies would be a moderate or major barrier. In conclusion, the intervention was well accepted by women and their families and feasible for government health staff within the context of a trial. Future scale up of FCM IV iron would require out-of-pocket costs to be covered by public funding, staff training on the intervention, and strategies, such as dedicated staff or task shifting, to allow for monitoring during and after the infusion.

## Introduction

Anemia during pregnancy is associated with adverse maternal and birth outcomes, including premature birth, low birth weight, and maternal mortality [1,2]. Anemia in pregnancy, blood loss during delivery, and inadequate iron supplementation during the postpartum period are significant determinants of postpartum anemia [3]. Globally, more than 40% of pregnant women are affected by anemia [4], with iron deficiency anemia as the main etiology in low- and middle-income countries (LMICs). South Asia has the highest burden of anemia worldwide [5]. Despite several national initiatives, the burden of anemia among women of reproductive age in India increased from 53% in 2015–2016 to 57% in 2019–2021 [6,7]. In India, 52% of pregnant women and, by some estimates, more than 75% of postpartum women are anemic [7,8].

Since 1970, India has implemented anemia prevention and control programs. The most recent national program, Anemia Mukt Bharat, was launched in 2018 and includes the provision of daily iron-folic acid (IFA) tablets for pregnant women starting from the fourth month of pregnancy and continuing through 180 days postpartum [9]. Nearly 70% of women report gastrointestinal intolerance, including nausea, constipation, diarrhea, indigestion, and metallic taste associated with IFA use, which likely affects compliance [10]. In addition to IFA tablets, another treatment option for anemia during pregnancy and the postpartum period is intravenous (IV) iron. Ferric carboymaltose (FCM) can be provided as a single IV iron infusion and has a larger increase in hemoglobin and ferritin levels, along with a favourable safety profile and fewer adverse effects, compared to iron sucrose [11–13]. A 2-arm randomized controlled trial, Prevention of Iron Deficiency Anemia Post-Delivery (PRIORITY) trial, was conducted in eight sites across seven countries to evaluate the effectiveness of single dose FCM IV iron versus oral iron in reducing anemia among postpartum women diagnosed with moderate anemia (Hb 7.0 to 9.9 g/dL) [14].

To provide complementary evidence on facilitators and barriers to FCM IV iron implementation in high-burden anemia settings, we conducted an implementation research (IR) sub-study alongside the PRIORITY trial in the Belagavi, India study site [15]. The aims of this IR study were to gather information on IV iron intervention characteristics, implementation settings, implementer characteristics, and the implementation process and to assess the feasibility, acceptability, fidelity, and cost and perceptions of cost of IV FCM implementation. The results of the PRIORITY IR study in Belagavi together with findings from one other PRIORITY IR site in Pakistan and from a hybrid effectiveness-implementation trial in postpartum women with moderate or severe anemia in Nigeria [16,17] will be reviewed to inform global guidelines on FCM IV iron use and roll-out of the intervention, if it is deemed effective.

## Methods

### Study design

This mixed-methods study used a convergent parallel design with quantitative and qualitative data collected concurrently [18]. The study was designed to measure selected Consolidated Framework for Implementation Research (CFIR) constructs and Proctor’s framework elements related to the use of a single infusion of FCM IV iron [19–21]. Quantitative methods included surveys and observations, and qualitative methods included in-depth interviews. The surveys were used to obtain data from a large sample, while the qualitative data were used to provide information on how and why participants made choices or behaved as reported. The Good Reporting of a Mixed Methods Study checklist is included in S1 Table.

### Study sites and participants

We conducted this study in the three sub-district hospitals (Hukkeri, Bailhongal, and Gokak) in Karnataka State where the PRIORITY trial was implemented. These are secondary level government facilities providing maternity, surgical, and other services to a population of 80,000–120,000 [22]. Hukkeri and Bailhongal hospitals have an average of 100 deliveries per month, whereas Gokak has 400–500 deliveries per month.

Differences in resources available in the three study hospitals during the trial and sub-district hospitals under usual conditions are shown in S2 Table. In brief, the hospitals participating in the trial had a supply of FCM IV iron and 10 ml syringes, a protocol for administering FCM IV infusion was in place, staff were trained to provide the infusions and supervised, a separate room for infusion was identified in each hospital, and an emergency kit for drug reactions was available. Most of these resources are not available in usual Indian sub-district hospitals, although hospitals may have IV iron sucrose that they procure with their own budgets and provide supervision for its administration, and 10 ml syringes may be available, but not consistently.

In the three study facilities, women had their hemoglobin measured within 48 hours postpartum, and if moderate anemia was established (7 to 9.9 g/dL), they were invited to participate in the PRIORITY trial and randomized to either oral iron or a single infusion of FCM IV iron. Less than 1% of the women who were eligible for the PRIORITY trial in India refused to participate.

Three groups of participants were included in the PRIORITY IR study: (1) moderately anemic postpartum women who participated in the IV iron study arm of the PRIORITY trial, (2) family members of women who received IV iron, and (3) government health workers who administered IV iron as well as hospital administrators involved in the trial.

Women in the PRIORITY trial were eligible for the IR study if they met these criteria: recently delivery, aged 18–45 years, confirmed moderate anemia, no contraindications to iron supplementation, had not received and were not scheduled to receive a blood transfusion, and consented to participate in the PRIORITY trial and IR study. Women who delivered in the three hospitals were systematically screened and those who were anemic were invited to participate in the trial and the IR study. All women randomly assigned to the IV iron arm in the trial agreed to participate in the implementation research. Family members, including husbands, mothers, mothers-in-law, and sisters, of women in the IV iron arm of the PRIORITY trial were eligible if they were 18 years or older and consented to participate in the IR study. Government health workers volunteered to be part of the PRIORITY trial. Health workers were eligible for the IR study if they were involved in administering IV infusions as part of the PRIORITY trial and hospital administrators were eligible if they were involved in overseeing the trial; they had to be 18 years or older and consent to participate in the IR study. All government health workers directly involved in implementing the IV iron intervention and hospital administrators connected to the trial were enrolled.

### Data collection

We conducted a survey with 308 moderately anemic postpartum women who had received a single dose of FCM IV iron post-delivery as part of the PRIORITY trial. The survey was administered after receipt of the IV iron infusion, either while the woman was still in the hospital or at home. Women who received IV iron were invited to participate in in-depth interviews (IDIs) until five IDIs per facility were completed (total N = 15). Additionally, fifteen IDIs (5 per facility) were conducted with family members ofwomen who had received IV iron post-delivery. Family member IDIs were limited to close relatives who accompanied women receiving IV iron in the PRIORITY trial, were actively involved in decision making, and present during the IV iron infusion. Family members were identified by nursing officers in the facilities, then approached by the study team to confirm eligibility and obtain consent. IDIs with women and family members were completed at the facility or at home. Surveys and IDIs were conducted with six hospital administrators, two from each facility. Ten surveys, and nine IDIs, were conducted with nursing officers (subsequently referred to as health workers) who had administered IV iron as part of the PRIORITY trial. All the hospital administrators and health workers were approached by the study team and those who provided consent were interviewed at the hospitals. Qualitative sample sizes were determined a priori in accordance with established qualitative research practices and were deemed sufficient to achieve data saturation [23].

Experienced data collectors participated in a training on the PRIORITY IR study design, purpose, study procedures, data collection tools, and research ethics. All tools were pilot tested and refined before starting data collection. Final versions of the tools are found in S3 File. Survey tools were programmed into REDCap and data were entered by the data collectors who administered the surveys to participants. IDIs were conducted by two researchers (UC and AD). Surveys and IDIs were conducted in Kannada or English, based on the participant’s preference. IDIs lasted 30–90 minutes and were audio recorded.

Data collection occurred from October 5, 2023 to June 28, 2024. Electronic data were stored on password-protected tablets or computers, and consent forms were stored in a locked cabinet within the Women’s and Children Health Research Unit, Jawaharlal Nehru Medical College in Belagavi, Karnataka, India. Audio recordings were given unique participant IDs and translated or transcribed verbatim into English. Personal identifiers were removed to protect confidentiality.

All participants signed written informed consent forms. Women and family members who participated in IDIs were reimbursed 250 Indian rupees. The study was approved by the following institutional review boards: Thomas Jefferson University institutional review board (March 25, 2023; iRISID-2023–1605); Institutional Ethics Committee, KLE (Karnataka Lingayat Education Society) Academy of Higher Education and Research, Belagavi, India (May 24, 2023; KAHER/EC/2023–24/d-24052307); and Research Triangle Institute (July 28, 2023; 12940-9a).

### Study variables

Surveys were used to collect participants’ demographic data, such as age, sex, education, and employment, and obtain data that aligned with selected constructs from CFIR domains and Proctor’s implementation outcome measures and implementation strategies (Fig 1). When the study was designed, the updated CFIR 2.0 constructs [20] were not available, so CFIR 1.0 constructs were used [19]. Selected CFIR constructs were chosen based on their relevance to our research questions, salience to the intervention, and contextual fit. Data on CFIR and Proctor’s variables were also collected through qualitative interviews. Information about cost per gram of FCM and participants’ perceptions of costs are included in this analysis. Cost effectiveness of the intervention will be reported separately for all PRIORITY trial sites.

**Fig 1:**
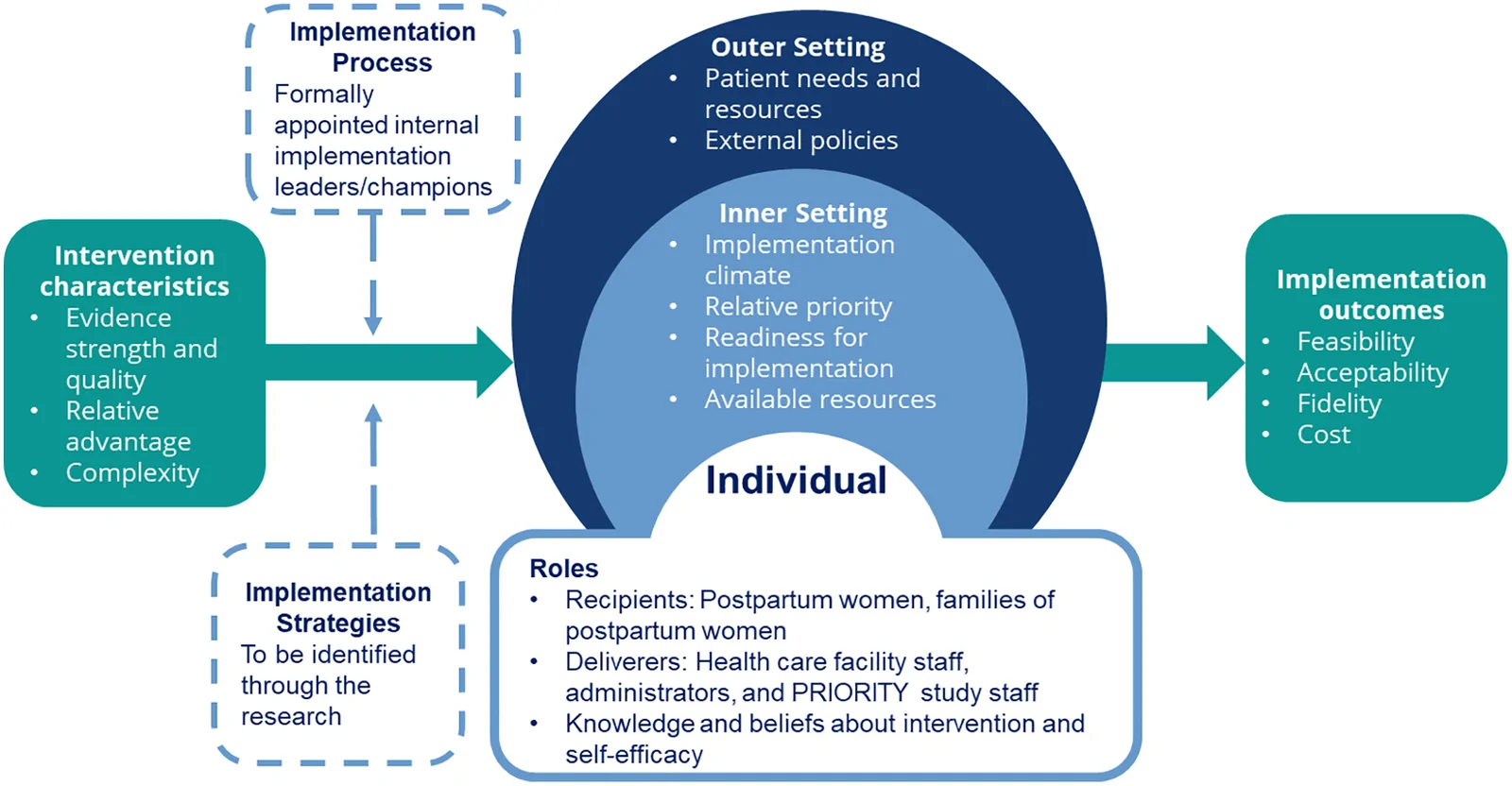
CFIR domains and constructs and Proctor’s implementation outcomes used in the PRIORITY IR study. Source: Flax VL, Rizvi N, Charantimath U, Jessani S, Kavi A, Saleem S, et al. PRIORITY IR: Protocol for implementation research on single-dose postpartum IV iron to treat iron-deficiency anemia among women in India and Pakistan. Gates Open Res. 2025;9:48.Data analysis.

### Data analysis

Quantitative data from surveys and observations were analyzed descriptively using SAS statistical software (version 9.4, SAS Institute, Cary, NC, USA) and reported as frequencies and percentages. The main analysis was performed for the combined data from the three hospitals. The Jawaharlal Nehru Medical College team from India (UM and AD) and Thomas Jefferson team (LW, SL, ST, RF), Philadelphia, USA analyzed the qualitative data with NVivo software (version 14.23.4, Lumivero, Denver, CO, USA) using directed content analysis methods [24]. All interviews were coded independently by two coders and differences in code application were discussed until consensus was reached. Codebooks were based on deductive codes using the CFIR and Proctor’s framework categories. The coded data were used to create data matrices [25].

Following the convergent parallel design model, quantitative and qualitative data were analyzed separately and then compared to find similarities and differences. Code reports for CFIR constructs and Proctor’s outcomes from the qualitative analysis were compared with quantitative results for the same constructs and outcomes. The analysis team discussed the few discrepancies between quantitative and qualitative findings and came to consensus on which quotes fairly represented the main findings.

The qualitative research team recognized that conducting the IR study within an ongoing clinical trial could shape perspectives on the data and its interpretation. To promote reflexivity, researchers engaged in regular discussions about their positions, assumptions, and potential biases throughout the study [26]. The multidisciplinary nature of the team and their varying levels of involvement with the trial led to critical examination of emerging interpretations. Some members of the analytic team were not involved in the trial design or implementation, providing independent perspectives during analysis.

## Results

This section describes participant characteristics, results related to CFIR domains and Proctor’s implementation outcomes, and a summary of convergence and divergence of the quantitative and qualitative findings.

### Participant characteristics

A total of 339 participants were enrolled in the study: 308 women who received IV iron, 16 health workers and hospital administrators, and 15 family members of the women. Sociodemographic characteristics of participants are shown by participant type and data collection method in Table 1. Postpartum women (N = 308) who received IV iron were enrolled for the IR survey across the three study hospitals as follows: Gokak (41%), Bailahongal (30%), and Hukkeri (29%). The mean age of postpartum women was 23 years and more than 90% of the woman had 7 or more years of schooling. This was the first pregnancy for approximately 45% of the women. A subset of 15 of these women participated in IDIs.

**Table 1:** Participant characteristics.

Participant Characteristics	n (%)
Women who received IV iron (survey)	N = 308
Site location	n (%)
Hukkeri	88 (28.6)
Bailahongal	93 (30.2)
Gokak	127 (41.2)
Age, years (mean, SD)	23.0 (3.5)
Education	
No formal school	8 (2.6)
1 - 6 years of schooling	20 (6.5)
7 - 12 years of schooling	240 (77.9)
≥13 years of schooling	40 (13.0)
Parity, including current pregnancy	
1	139 (45.1)
2	105 (34.1)
≥3	64 (20.8)
**Women who received IV iron (in-depth interviews)**	**N = 15**
Site location	n
Hukkeri	4
Bailahongal	4
Gokak	7
Age, years (mean)	28
Education	
1 - 6 years of schooling	1
7 - 12 years of schooling	5
≥13 years of schooling	9
Parity, including current pregnancy	
1	10
2 or more	5
**Health workers and hospital administrators (survey)**	**N = 16**
Site location	n
Hukkeri	5
Bailahongal	5
Gokak	6
Job title	
Nursing officer	10
Administrator	6
Years worked at job (mean, SD)	9
**Health workers and hospital administrators (in-depth interviews)**	**N = 15**
Site location	n
Hukkeri	5
Bailahongal	5
Gokak	5
Years worked at job (mean, SD)	13
**Family members (in-depth interviews)**	**N = 15**
Relationship with participant	n
Mother	8
Sister	2
Sister-in-law	2
Mother-in-law	1
Father	1
Husband	1

Ten health workers and six hospital administrators (total N = 16) were enrolled in the study. They were divided across the study hospitals as follows: Gokak (6), Bailahongal (5), and Hukkeri (5). They had worked in their positions for an average of 9 years (range 3–30 years). All of the health workers were nursing officers. Fifteen of the health workers and hospital administrators were included in IDIs. One of the health workers did not participate in an IDI because she was on an extended leave due to a health condition.

A total of 15 family members were enrolled in IDIs. Their relationship to the woman who received IV iron was: mother (8), sister (2), sister-in-law (2), mother-in-law (1), father (1), and husband (1).

### CFIR domains

Health workers’ and hospital administrators’ survey responses related to CFIR domains and selected constructs are shown in Table 2 and a breakdown of their responses by hospital is found in S4 Table.

**Table 2:** Survey findings from health workers and hospital administrators related to CFIR intervention characteristics, outer setting, inner setting, and individual domains.

CFIR Domains and Constructs	Health Workers and Hospital Administrators n
Intervention characteristics	N = 16
Evidence strength and quality	
Sources of information about FCM IV iron for postpartum women with anemia	
Published literature	5
Co-worker experience	3
Practice guidelines	9
Patient experience	8
PRIORITY trial training	14
Doctor’s advice	4
Other	2
How this information affects your confidence about providing FCM IV iron	
I am more confident providing IV iron	13
I am less confident providing IV iron	2
I am neutral providing IV iron	1
Relative advantage	
Is FCM IV iron or oral iron better in your opinion	
Yes, oral iron is a better treatment than IV iron	2
Yes, IV iron is a better treatment than oral iron	13
No, one treatment is not better than the other	1
Advantages of FCM IV iron compared to oral iron	
Effective for most patients	8
More effective than oral iron	9
Patient compliance is assured	14
Patient has one visit	10
Ability to administer large dose in single infusion	12
Little or no side effects	1
Disadvantages of FCM IV iron compared to oral iron	
No disadvantages	4
Adverse reactions	12
High cost	7
Requires trained and skilled staff	5
Requires equipment	3
Requires time to administer	4
Requires monitoring IV infusion	4
Availability of emergency drugs for adverse events	1
Complexity	
Challenges in providing FCM IV iron to postpartum women with moderate anemia	
No challenges	4
Length of time to administer IV iron	6
Number of steps to follow	7
Length of time to train workers to administer IV iron	2
Not enough trained or skilled workers to administer IV iron	8
Woman needs to be monitored constantly	7
Ability to manage adverse events during or after infusion	8
Availability of emergency drugs for adverse events	3
Woman or family needs convincing	4
Woman needs support	1
Providing FCM IV iron is more difficult for me as a health worker than giving oral iron to postpartum women (Health workers only, N = 10)	
Strongly disagree	4
Disagree	1
Undecided	1
Agree	3
Strongly agree	1
How easy or difficult is it to administer FCM IV iron compared with providing other services	
Very difficult	0
Difficult	1
Neutral	3
Easy	11
Very easy	1
**Outer setting**	**N = 16**
Patient needs and resources	
What are the needs and preferences of postpartum women related to anemia treatment	
They prefer IV iron	5
They prefer oral iron	7
They prefer both IV iron and oral iron	0
They prefer nutrition counseling	3
Other	1
Before the PRIORITY trial, how aware were you/hospital staff of the needs and preferences of postpartum women with moderate anemia	
Not aware at all	1
Slightly aware	1
Somewhat aware	1
Moderately aware	8
Extremely aware	5
The needs and preferences of postpartum women served by this hospital were considered before implementing the PRIORITY trial	14
How well do you think the FCM IV iron used in this trial meets the needs of postpartum women with moderate anemia	
Not well at all	0
Slightly well	1
Somewhat well	1
Moderately well	3
Extremely well	11
Barriers postpartum women face to receiving IV iron	
No barriers	3
Fear of injection	7
Cost	3
Think it is ineffective	2
Concern of side effects to self	9
Concern of side effects to child	6
Other	6
External policies (Administrators only)	**N = 6**
What factors influenced the decision to implement the PRIORITY trial in this hospital	
Nothing influenced the decision	1
Hospital was approached to participate in the trial	4
Local policies	3
State policies	3
National policies	2
Hospital clinical performance measures	1
Financial incentive	0
Other	3
**Inner setting**	**N = 16**
Relative priority	
How much of a priority is FCM IV iron for postpartum women compared with other health services	
Not a priority	0
Low priority	0
Medium priority	2
High priority	11
Essential	3
Readiness for implementation	
How open are you/hospital staff to providing FCM IV iron to postpartum women after the PRIORITY trial ends	
Not at all receptive	0
Slightly receptive	0
Somewhat receptive	0
Moderately receptive	3
Extremely receptive	13
Available resources	
The hospital has sufficient resources to implement and administer FCM IV iron after the trial ends	7
After the PRIORITY trial ends, how much of a barrier will procuring necessary resources be to providing FCM IV iron to women postpartum	
Not a barrier	2
Somewhat of a barrier	5
Moderate barrier	6
Extreme barrier	3
**Individual**	**N = 16**
Knowledge and beliefs about intervention	
How knowledgeable are you about FCM IV iron implementation	
Not at all knowledgeable	0
Slightly knowledgeable	1
Somewhat knowledgeable	0
Moderately knowledgeable	7
Extremely knowledgeable	8
Self-efficacy	
How confident are you/hospital staff can successfully administer FCM IV iron to postpartum women	
Not confident at all	0
Slightly confident	0
Somewhat confident	1
Fairly confident	2
Completely confident	13

### Intervention characteristics

The constructs measured under intervention characteristics included evidence for the strength and quality of the intervention, relative advantage of the intervention, and intervention complexity (Table 2).

#### Evidence strength and quality.

Based on the survey, most health workers and hospital administrators had obtained information about how to administer FCM IV iron through PRIORITY trial training (14/16), practice guidelines (9/16), or patient experience (8/16), but very few mentioned having information about the efficacy of FCM. For example, one health worker explained that they learned about FCM from the trial training.

*“No, I have not heard about it [FCM IV iron] before coming here. I have heard about infusing iron sucrose only. After coming into this project, I heard about it [FCM IV iron].” – Health worker*

Another health worker mentioned that they heard about FCM from practice guidelines and coworkers.

*“From practice guidelines, even the literature, and also our experience, and we do discuss about everything with coworkers**…** [I heard from coworkers] that FCM is safe and hardly anybody has encountered any adverse reactions, other than mild rashes.” – Health worker*

#### Relative advantage.

In the survey, most health workers and hospital administrators (13/16) said that FCM IV iron is a better treatment than oral iron in their opinion, while 2/16 said that oral iron is better. The most frequently cited advantages of FCM IV iron compared to oral iron were that patient compliance is assured (14/16), a large dose could be administered in a single infusion (12/16), and the patient only required one visit (10/16). A health worker explained why these advantages are important.

*“As far as oral iron is concerned, women forget to take oral iron…They will not take those tablets after going home. They neglect taking them…and they sometimes throw away the tablets, whereas this FCM IV iron is only a one-time infusion. Once it is infused, it is over and after two months or three months the blood will improve, I think, so it is good.” – Health worker*

Another health worker explained why FCM is better than oral iron and IV iron sucrose.

*“By infusing FCM, this will improve their blood. They will not be having any mental tension to take any iron, calcium, and nutrition tablets. We have to give iron sucrose three days in a row. But this FCM, gives us the result through one infusion on one day.” – Health worker*

The most frequently cited disadvantages of FCM IV iron compared to oral iron were the possibility of adverse reactions (12/16), high cost (7/16), and administration of FCM IV iron requiring trained and skilled staff (5/16). For example, one health worker said, *“You need trained sisters [nurses], you need equipment, you need to maintain the emergency drugs, and everybody has to be trained to address the adverse effects, then it is also time consuming.”*

#### Complexity.

In the survey, the complexity or challenges with providing FCM IV iron that health workers and hospital administrators cited most frequently were the lack of trained or skilled workers to administer it (8/16), ability to manage adverse events during or after infusion (8/16), the number of steps that they have to follow to administer FCM (7/16), and the need to monitor the woman constantly during infusion (7/16). Health workers were divided on whether providing FCM IV iron to women is more difficult for them compared to giving them oral iron. Four out of 10 health workers agreed or strongly agreed that FCM IV iron is more difficult to provide, whereas five out of 10 disagreed or strongly disagreed. Compared with providing other services, health workers and hospital administrators said administering FCM IV iron was easy or very easy (12/16). Health workers explained why monitoring the patient after the FCM IV iron infusion was difficult for them and how the challenges with providing FCM IV iron could be addressed through training and providing more staff or staff dedicated to this task. Health workers mentioned the following related to complexity:

*“The observation part is difficult for us. In this project, you observe for half an hour. But here, it is difficult for us to observe for half an hour. We always move from one place to another in the hospital. It is good if some staff [are] posted only for that work [of providing FCM IV iron infusions].” – Health worker*

*“It is not a problem to address these challenges. If the staff is well trained, it can be done. It is okay if trainings are conducted repeatedly, like twice in a year. The skills also will be improved.” - Health worker*

### Outer setting

The constructs measured for outer setting included patient needs and resources and external policies (Table 2).

#### Patient needs and resources.

Based on the survey, health workers and hospital administrators were divided on what they thought the needs and preferences of postpartum women were related to anemia treatment, with 7/16 saying that women prefer oral iron, 5/16 saying they prefer IV iron, and 3/16 indicating they prefer nutrition counselling. Most health workers and hospital administrators said they were moderately or extremely aware of the needs and preferences of postpartum women with anemia before the PRIORITY trial (13/16), the needs and preferences of women were considered before starting the trial (14/16), and FCM IV iron used in the trial meets the women’s needs moderately or extremely well (14/16). The most frequently mentioned barriers that women face in receiving IV iron postpartum were women’s concern of side effects to themselves (9/16), their fear of injections or needles (7/16), and their concern of side effects to the child (6/16). A hospital administrator described how some women were initially worried about the side effects of FCM IV iron, but when health workers provided them with more information, they almost always agreed to receive the infusion.

*“Women, they are mainly worried about the IV drug, not the oral drug. So, acceptance of IV compared with oral is less among the public.... When we counsel them about the adverse reactions, they step back. That is the only drawback. However, when we tell them that the adverse effects are very minimal with this drug, the acceptance is there and hardly any women have refused the infusion.” – Hospital administrator*

#### External policies.

Administrators were surveyed regarding the policies and other factors that influenced the decision to implement the PRIORITY trial in their hospital. Most mentioned that the hospital was approached to participate in the trial (4/6). Although there are currently no policies related to FCM IV iron, an administrator explained their thoughts about the next steps the government should take in creating a policy if the intervention proves effective.

*“As I have seen, the results are really good with the FCM IV iron and after this trial, I feel personally, it has to be made available in the government hospitals. And I think the government is capable enough to make it happen…If it is going to help the mothers, the government obviously is giving so much importance to mother and child health, this is one thing that has to be implemented.” – Hospital administrator*

### Inner setting

The constructs measured for inner setting included relative priority, readiness for implementation, and available resources (Table 2).

#### Relative priority.

Based on the survey, health workers and hospital administrators felt that FCM IV iron for postpartum women was a high priority (11/16) or essential (3/16) compared to other health services. One hospital administrator explained why they think FCM IV iron is a priority for postpartum women.

*“Actually, we give first priority to women in their postnatal period. Since it is the life of two people, the baby and the mother, in the postpartum period, the mother should be healthy to feed the baby. So, I feel that, compared to other services in the hospital, the first priority has to be given to the postpartum women with moderate anemia.” – Hospital administrator*

#### Readiness for Implementation.

The majority of health workers and hospital administrators thought that their hospital was extremely receptive (13/16) to providing FCM IV iron to postpartum women after the PRIORITY trial ends.

*“It can be implemented. We have already selected the designated areas, and we have arranged emergency drugs and resuscitation equipment. Except the molecule [the FCM compound], we have everything... [but] sometimes the cost might be a challenge.” – Hospital administrator*

#### Available resources.

Less than half of health workers and hospital administrators (7/16) thought their hospital had enough resources to implement FCM IV iron after the PRIORITY trial ends. More than half of them (9/16) reported that procuring resources to provide FCM IV iron to women postpartum would be a moderate or extreme barrier to implementation. A hospital administrator explained that they have most of the necessary supplies and equipment, but they are lacking access to FCM and the ability to pay for it, unless it is provided through the government system.

*“We need IV fluids, drip sets, cannula, easy fix, emergency drugs trays, a timer, gloves. We have all of these…except FCM. We need FCM.” – Hospital administrator*

*“This [FCM] is more costly. Therefore, our other purchases will be affected to a certain level, and it will be a problem for us.” – Hospital administrator*

### Individual characteristics

The constructs measured for individual characteristics included knowledge and beliefs about the intervention and self-efficacy (Table 2).

#### Knowledge and beliefs.

Most health workers and hospital administrators rated themselves as moderately (7/16) or extremely (8/16) knowledgeable about FCM IV iron implementation. Women who received IV iron were asked about their knowledge and beliefs related to FCM IV iron. Nearly all of them (98%) said they believe that IV iron will reduce their anemia and make their symptoms better. More than two-thirds of the women (69%) said they were very likely to recommend single infusion IV iron to a family member or friend who needed it. Two women described their beliefs related to FCM IV iron and how it affected them as follows:

*“I feel that taking IV iron is better than taking tablets. If it had come as tablets for me, if I had taken tablets even from the beginning, my hemoglobin would not have increased. And with IV iron even if I take one dose, my hemoglobin would have increased. I feel that next time also I will take the same.” – Woman who received FCM IV iron*

*“I will feel that my blood will increase. It is possible to get up and walk around. I can* *breastfeed the baby better by sitting up properly, and I can sense giddiness reducing.” – Woman who received FCM IV iron*

#### Self-efficacy.

The majority of health workers and hospital administrators were completely confident (13/16) that they or their hospital staff can successfully deliver FCM IV iron to postpartum women. A health worker explained that they feel more confident about providing the FCM IV iron infusion after being trained and providing it to women as part of the PRIORITY trial.

*“There is a change. Now I have more courage that we also can provide this infusion if it is introduced in the government system. I have more courage now, but we still need a doctor with us (laughs).” – Health worker*

### Implementation process

The construct measured for the implementation process was related to formally appointed internal implementation leaders. Health workers explained that there were no appointed implementation leaders under this trial, but that the administrator, chief medical officer, other doctors, and nurses would need to be on board to roll out implementation of FCM IV iron in their hospitals.

*Interviewer: Has this hospital appointed an implementation leader a champion for FCM IV iron?*
*Health worker: No*
*Interviewer: Which leaders or individuals are influential in the hospital who need to be on board for the implementation of FCM IV iron to be successful?*
*Health worker: Yes, first thing is our CMO (chief medical officer), then the gynecologists who are working here, and even our staff nurses.*

### Proctor’s implementation outcomes

Health workers’, hospital administrators’, and women’s survey responses related to acceptability, feasibility, and perceived cost are shown in Table 3, a breakdown by hospital of acceptability and feasibility results from health workers and hospital administrators is found in S4 Table, and observation results related to fidelity of implementation are shown in Table 4.

**Table 3:** Survey Findings from health workers, hospital administrators, and women who received IV iron on proctor’s acceptability, feasibility, and cost implementation outcomes.

Proctor’s Implementation Outcomes	
Acceptability	
Health workers and hospital administrators	N=16
Providing FCM IV iron to postpartum women is acceptable to me	n
Completely disagree	0
Disagree	0
Neither agree nor disagree	0
Agree	4
Completely agree	12
I have no objection to providing FCM IV iron to postpartum women	
Completely disagree	0
Disagree	0
Neither agree nor disagree	0
Agree	3
Completely agree	13
**Women who received IV iron**	**N=308**
Receiving IV iron met participants’ expectations	n (%)
Completely disagree	2 (1)
Disagree	0 (0)
Neither agree nor disagree	28 (9)
Agree	110 (36)
Completely agree	168 (54)
Compared to receiving oral iron, receiving IV iron was appealing to the participant	
Completely disagree	1 (0)
Disagree	2 (1)
Neither agree nor disagree	67 (22)
Agree	111 (36.0)
Completely agree	127 (41)
Receiving IV iron was better than expected	
Completely disagree	1 (0)
Disagree	1 (0)
Neither agree nor disagree	48 (16)
Agree	126 (41)
Completely agree	132 (43)
Participant would agree to receive IV iron again if needed	
Completely disagree	3 (1)
Disagree	8 (3)
Neither agree nor disagree	33 (11)
Agree	87 (28)
Completely agree	177 (57)
**Feasibility**	
**Health workers and hospital administrators**	**N=16**
Providing FCM IV iron to postpartum women seems implementable	n
Completely disagree	0
Disagree	1
Neither agree nor disagree	0
Agree	10
Completely agree	5
Providing FCM IV iron to postpartum women seems easy	
Completely disagree	0
Disagree	0
Neither agree nor disagree	3
Agree	6
Completely agree	7
**Women who received IV iron**	**N=308**
Participant feeling regarding the process of IV iron infusion	n (%)
Very difficult	0 (0)
Somewhat difficult	12 (4)
Neither difficult, nor easy	10 (3)
Somewhat easy	49 (16)
Very easy	237 (77)
Participant concerns about receiving IV iron before receiving it	
No concerns	235 (76)
Afraid of having an infusion/needles	40 (13)
Not sure it would work	5 (2)
Afraid of side effects	22 (7)
Other	14 (5)
Participant still has concerns (n=73)	20 (27)
Barriers participant faced in receiving IV iron	
No barriers	303 (98)
Concern about the safety of the drug	0 (0)
Financial constraints for staying in the hospital	0 (0)
Family did not want participant to receive IV iron	0 (0)
Family was not available to discuss receiving IV iron	0 (0)
Other	5 (2)
**Perceived Cost**	
**Women who received IV iron**	n (%)
Participant is willing to pay for IV iron treatment ‘out of pocket’ if IV iron is needed in the future (N=308)	227 (74)
Amount willing to pay (mean, SD) (N=227)	1393(2408)

**Table 4:** Observation of health workers for fidelity of implementation (n = 45 observations).

Fidelity of Implementation	n (%)
Infusion Set & Participant	
The healthcare provider checked if there is any evidence of damage to the IV infusion set	45 (100.0)
If identified, the healthcare provider replaced the set and logged the issue	n/a
The health care provider inspected the IV Iron vial for visible precipitation or particulate matter	45 (100.0)
If identified, the healthcare provider replaced the vial and logged the issue	n/a
The healthcare provider recorded all vital signs on PT03	43 (95.6)
If there were irregularities in any of the vital signs, they were taken again to confirm whether they remained irregular	1/1 (100.0)
If they remained irregular, the healthcare provider used judgement regarding whether to delay or cancel the infusion	n/a
The healthcare provider did not give any kind of pre-medication before the administration of the IV iron drug	45 (100.0)
The healthcare provider counseled the participant about the procedure and what to expect	45 (100.0)
The healthcare provider answered any questions in a reassuring manner	3/3 (100.0)
**Administration of FCM IV Iron**	
The healthcare provider started the normal saline first	45 (100.0)
The healthcare provider then added the IV iron into normal saline bottle	45 (100.0)
The healthcare provider inverted the IV iron bag after iron was added to the infusion bag	45 (100.0)
The healthcare provider started the timer to monitor the infusion when the health care provider saw the drug enter the cannula	45 (100.0)
The healthcare provider closely observed the participant for 5 minutes before increasing the infusion administration rate	45 (100.0)
The healthcare provider completed the infusion within 25–30 minutes	44 (97.8)
**Monitoring**	
Two healthcare providers were in the room with the participant to monitor the infusion at all times	45 (100.0)
If there was any evidence that the participant was not tolerating the infusion, the healthcare provider immediately paused the IV iron infusion but left the cannula in place in case emergency medications were required	1/1 (100.0)
**During the IV Iron Infusion**	
The healthcare provider visually observed the participant for any side effects	45 (100.0)
The healthcare provider verbally assessed the participant by asking questions	45 (100.0)
The health care provider observed the IV line to ensure the drip had a consistent flow and the line was intact throughout the procedure	45 (100.0)
**After the IV Iron Infusion**	
The healthcare provider visually observed the participant for at least 30 minutes	45 (100.0)
The healthcare provider asked the participant how she was feeling	45 (100.0)
The health care provider kept the cannula in place until the 30-minute post-infusion monitoring period was completed in case emergency medications were required	45 (100.0)
If the participant was not comfortable after 30 minutes, the healthcare provider extended the monitoring period for another 30 minutes	4/4 (100.0)
**Discharge**	
The healthcare provider showed the participant the contents of the folic acid box while reviewing the Discharge Handout	45 (100.0)
The healthcare provider asked the participant to remove one folic acid pill and take it	45 (100.0)
The healthcare provider watched the participant take pill (and ensured that water or other drink was available)	45 (100.0)
The healthcare provider recorded pill consumption in drug accountability logbook	41 (91.1)
After the participant took the pill, the healthcare provider returned the blister strip to the folic acid box and asked participant to take box with her	44 (97.8)
The healthcare provider reminded the participant that she should take another pill tomorrow	45 (100.0)

#### Acceptability.

Surveys showed that all (16/16) health workers and hospital administrators agreed or completely agreed that providing FCM IV iron to postpartum women is acceptable to them and they had no objection to providing it, indicating a high level of acceptability (Table 3). Surveys with women who received FCM IV iron also showed that the intervention was acceptable. Most women agreed or completely agreed that receiving IV iron met their expectations (90%), receiving IV iron was appealing compared with receiving oral iron (77%), receiving IV iron was better than expected (84%), and they would agree to receive it again if needed (86%).

*“Some [women] do feel that it is better if you give oral [iron]…But when they have met the IV iron criteria, we describe the good or the positive aspects of FCM, like single shot and not needing to take tablets for six months where you can forget the tablets, then they do accept IV infusion. You need to educate them, then acceptance is very high.” – Hospital administrator*

Family members generally described the experience of the woman receiving the FCM IV iron infusion as very acceptable.

*“I felt that they did all that with so much confidence. I thought that they had the confidence in doing their job by looking at the way they were infusing the iron to my daughter. They infused the IV iron very carefully so that my daughter did not feel any kind of discomfort…They came to the room to check the blood pressure and talked to my daughter and asked whether she was feeling comfortable or not.” – Family member*

#### Feasibility.

Surveys with health workers and hospital administrators indicated that most of them agreed or completely agreed that providing FCM IV iron to postpartum women seems implementable (15/16) and easy (13/16) (Table 3). Surveys with women who received FCM IV iron found that most women felt the process of the IV iron infusion was very easy (77%), they had no concerns about receiving IV iron before they got it (76%), and they faced no barriers to receiving IV iron through the trial (98%). Those who did have concerns before receiving IV iron were mainly afraid of the infusion/needles or worried about side effects. For example, one woman explained that she was initially afraid of the needle, but once the infusion was started, she was not afraid, so it seemed more feasible.

*“First, I had the fear that they would prick me with the needle again. After that, I did not have any fear. They also told me that the saline would be infused slowly, and I did not feel any fear.” – Woman who received IV iron*

Another woman talked about how the injection is feasible because it is done through one infusion, whereas oral iron requires taking the tablets regularly, which can be difficult.

*“Injection group means, we need to get it infused once...That is all. For [oral iron] tablets, we have to take them continuously for six months...Sometimes we may forget to take the tablets, sometimes we do not want to take, and sometimes it causes vomiting. Therefore, we cannot take tablets regularly.” – Woman who received IV iron*

#### Cost and perceived cost.

At the time of the trial, the cost of FCM IV iron was 8000 Indian rupees (98 USD) per gram. The price is currently 2000 Indian rupees (24 USD) per gram. Most women receive a one gram dose of FCM based on their weight. Women received FCM for free as part of the trial. Women and their family members were not aware of the price of FCM because it was not available outside of the trial, but they were asked how much they would be willing to pay and their perceptions of the cost. Most women who received IV iron (74%) stated during the survey that they would be willing to pay for FCM IV iron out of pocket if they need it in the future (Table 3). The amount they could pay on average was 1392 Indian rupees (17 USD). However, the qualitative data indicated that if women or their families had to pay the full amount themselves, it would be challenging for them.

*“I will receive it, but if it is free. We can pay if it costs rupees five hundred or a thousand, but we may not be able to afford more than that.” – Woman who received IV iron*

*“If it is from the government, half of the expenditure would be borne by the government and half of the cost can be borne by us.” – Family member*

#### Fidelity.

Table 4 shows results from observations of health workers carrying out FCM IV iron infusions as part of the PRIORITY trial. Health workers correctly performed nearly all steps in setting up the infusion, administering it, monitoring the patient during and after the infusion, and discharging the patient. The minor exceptions were recording all vital signs on the correct form (96%), completing the infusion in 25–30 minutes (98%), and recording folic acid pill consumption by the patient in the drug accountability logbook (91%).

#### Implementation strategies.

Four key strategies for implementing FCM IV iron for postpartum women that were identified during this study included: establishing rapport with women and their accompanying family members; providing counseling on the advantages and possible adverse effects of FCM; making sure health workers are trained to provide FCM; and assigning specific health workers to this task or task shifting. The following quote from a family member shows how creating rapport and providing counseling helps with implementation.

*“They came to her bed and told that her blood had become very less…What I asked them was, “Does any problem occur when the IV is infused?” They told me that some people tolerate it, and some may not tolerate. After that, I removed any kind of confusion from my mind and told them to give the IV infusion.” – Family member*

### Convergence and divergence between qualitative and quantitative findings

S4 Table summarizes the quantitative and qualitative findings for each CFIR construct and Proctor’s outcome and indicates whether the results converged or diverged. There was generally good convergence between quantitative and qualitative results across CFIR constructs and Proctor’s acceptability and perceived cost outcomes. Results for feasibility converged across methods for the women who received IV iron, but responses from health workers and hospital administrators differed between Proctor’s feasibility outcome and the CFIR complexity construct. Health workers said that FCM IV iron was easy to implement (Proctor’s feasibility outcome), but mentioned several challenges to implementation, such as monitoring the woman for 30 minutes and skills required to provide the intervention (CFIR complexity construct).

## Discussion

This is one of the first studies to report on facilitators and barriers to the implementation of single infusion FCM IV iron in postpartum women using IR frameworks. Our use of mixed methods allowed us to gather structured quantitative data while complementing it with qualitative findings that helped explain the meaning of the survey results. In terms of facilitators, data collected from health workers, hospital administrators, postpartum women, and their family members confirmed a high level of acceptability and feasibility of the FCM IV iron intervention. Our observations of intervention implementation by health workers involved in the trial in the three hospitals showed a high level of fidelity to the implementation protocol. The PRIORITY trial team hypothesized that >80% fidelity would be needed to scale up and that was achieved in these hospitals in India and across all PRIORITY trial sites. Women reported very few barriers to receiving IV iron in this study. Hospital administrators said they would give a high priority to this intervention and would be ready to implement it after the trial, if they can get the FCM iron.

The primary potential barriers to scaling up the implementation of FCM in the Indian context include: the financial burden on hospitals for procuring FCM and the necessary resources to administer it; the out-of-pocket cost for patients if the treatment is not publicly funded; the requirement for adequate staffing and training to ensure health workers have the skills and time needed to monitor patients receiving FCM; and the need to integrate FCM use into national health policy. No implementation champions for FCM IV iron were identified at the facilities in this study because it was part of a clinical trial, but it may be important to designate champions if the intervention is adopted in new hospitals.

In India, oral IFA tablets are standard of care for treating anemia during pregnancy and postpartum. The national anemia policy also includes the use of IV iron sucrose as a treatment option for moderate to severe anemia but recommends four infusions with 2–4 days between doses [27]. Against this backdrop, most health workers and hospital administrators involved in this study previously knew about iron sucrose but learned about FCM during training associated with the PRIORITY trial. They felt that the ability to give FCM IV iron as a single dose was a key advantage because it assured adherence, which is often a problem with IFA because of side effects and difficulties women face in remembering to take the pills [28,29] and can be a challenge with IV iron sucrose because of the opportunity costs related to return visits for all infusions. Women and their family members were generally accepting of IV iron treatment and appreciated that FCM is given as a single dose that could quickly alleviate anemia symptoms, increase a woman’s energy levels, and facilitate her ability to take care of her baby. Qualitative studies related to FCM IV iron for pregnant women in Nigeria and Malawi had similar findings and underscored the advantages of FCM in terms of ease of adherence and the rapid improvement in symptoms as compared to oral iron [30,31].

Health workers and hospital administrators reported an appreciation for the benefits of FCM, but noted several concerns related to scale up, these included: potential adverse reactions to FCM IV iron; its high cost; and the need for skilled staff to deliver the treatment, monitor patients, and manage adverse events. Health workers felt that delivering FCM was harder than oral iron because of the time needed to screen the patient, provide the infusion, and then monitor them afterwards. Women who received FCM were monitored for the duration of the IV iron infusion and then for at least 30 minutes post-infusion to ensure safety, detect hypersensitivity reactions, and optimize iron repletion. The monitoring could be task shifted to less skilled staff, provided that appropriate medical personnel are available in case of severe adverse reaction. This could help to reduce the additional workload that skilled health workers in this study mentioned and could facilitate scaling FCM IV iron for moderately anemic women postpartum.

Most women who received IV iron in this study had no concerns about the intervention, but those who did were mainly afraid of having an infusion, needles, or of the risk of side effects. Only 5% of the women reported experiencing side effects and all of them received help from health workers to manage the side effects. None of the women reported that their family did not want them to receive FCM IV iron and the family members interviewed were supportive.

Health workers explained that patients’ concerns related to needles and side effects of FCM could be addressed by providing information and counselling before giving the IV iron. Studies on FCM IV iron in Nigeria and Malawi also identified health workers’ and patients’ fears about adverse events and patients’ fear of needles, indicating that these are common feelings about IV iron during pregnancy or postpartum across settings [30,31]. The importance of providing information to patients and their family members, especially those who have decision-making power, was also noted in both of those studies.

The cost of FCM IV iron was a concern for women and their family members. Although nearly three-quarters of the women said they were willing to pay for the treatment, the women and their family members mentioned that if the cost was too high, they could not afford it. Similarly, health workers and hospital administrators were worried about the cost of FCM and mentioned the importance of having FCM, if proven effective, included in the national anemia policy and the essential medicines list so that it could be provided to health facilities and offered to patients for free. The cost of FCM has decreased in India since this study was conducted, as new companies have entered the market with their products, which could improve affordability for private citizens and the government.

This study had a few limitations, some of which are related to this being a sub-study within a clinical trial. We could only assess how implementation worked within a trial context and could not test different implementation strategies. Fidelity of implementation was high, but this may be related to trial-specific training and supervision, and fidelity may be lower under usual hospital conditions where there may be fewer staff assigned to administer IV iron, insufficient space, and less support for the intervention. Although all government health workers and administrators involved in the study were included, the total number was small and data from more health workers across different parts of India would provide additional information to prepare for intervention adoption. In addition, health workers volunteered to be part of the PRIORITY trial, so their responses to implementation questions in the IR study could be different from health staff working under routine conditions. Use of survey questions to ask about willingness to pay within the context of a trial where the intervention was offered for free may be influenced by social desirability bias, although the low prices women quoted on average suggest this bias was minimal.

If FCM proves to be effective for treating anemia in postpartum women, information from this IR study about facilitators, barriers, and perceived effective implementation strategies could be useful for intervention scaling. It will be important to conduct training for hospital staff to increase their confidence and skills in giving FCM IV iron; assign specific staff to carry out the intervention; and develop task shifting strategies to limit the monitoring time burden on skilled staff. Counseling and building rapport with the woman and her family members is essential, especially if the family members play a key role in decision-making [32]. In addition, to overcome issues related to the cost and availability of FCM, a government policy would need to support the use of FCM, thereby allowing FCM to be supplied as a commodity available to hospitals. Future research could study FCM IV iron implementation among postpartum women outside the context of a trial to understand how it is implemented and what implementation strategies are used in usual health care settings.

## Supporting information

S1 Table Good Reporting of a Mixed Methods Study (GRAMMS) Checklist. (DOCX)

S2 Table Resources Available in Government Hospitals During the PRIORITY Trial Compared with Indian Sub-district Hospitals Under Usual Conditions. (DOCX)

S3 File Data Collection Tools. (PDF)

S4 Table Survey Findings from Health Workers and Hospital Administrators Related to CFIR Domains and Proctor’s Outcomes by Hospital. (DOCX)

S5 Table Summary of Quantitative and Qualitative Results for CFIR Constructs and Proctor’s Implementation Outcomes. (DOCX)
