# Supplementary material for: Using implementation research frameworks to identify facilitators and barriers to implementation of ferric carboxymaltose intravenous iron among moderately anemic postpartum women participating in a trial in India
Source: PLoS One. 2026 Oct 21;21(10):e0359787. doi: 10.1371/journal.pone.0359787 (PMC13686276; doi:10.1371/journal.pone.0359787)
Supplement: Supplementary file 1 [file pone.0359787.s003.pdf]

This form should be completed by the *study staff* for all women who enrolled in the IV iron arm of PRIORITY trial prior to their discharge from hospital after delivery. Complete before ending the exit survey for women enrolled in trial.

Section A. Introduction/Background

First, I will ask some questions about you and your experience with taking iron tablets and receiving IV iron.

NO.	QUESTION	RESPONSE CODE	SKIP
0	Please select the site location.	Jinnah Postgraduate Medical Center (JPMC) (Pakistan)……………………………………1<br>General Hospital, Bailhongal (Belagavi, India) ……………………………………………………2<br>General Hospital, Hukkeri (Belagavi, India) ……………………………………………………3<br>General Hospital, Gokak (Belagavi, India) ……………………………………………………4	
1	How old are you?	[__][__] AGE IN YEARS	
2	Did you take iron tablets during this pregnancy?	Yes .... 1<br>No 2	→ **B.Q1**
3	How many weeks did you take the iron tablets during this pregnancy?	[__][__] WEEKS OF IRON TABLET USE	
4	What were the benefits of taking oral iron tablets compared to IV iron?<br><br>DO NOT READ RESPONSES. MULTIPLE RESPONSES POSSIBLE.	No benefits……………………………………0<br>Tablets are easy to use .... 1<br>Tablets are inexpensive.................................. 2<br>Tablets are readily available .... 3<br>I have used oral iron before .... 4<br>I trust the tablets .... 5<br>I can take tablets at home................................ 6<br>Tablets are easier because I fear needles........ 7<br>Other (specify) .... 98<br>Don't know……………………………………99	
5	What were the challenges of taking oral iron tablets compared to IV iron?<br><br>DO NOT READ RESPONSES. MULTIPLE RESPONSES POSSIBLE.	No challenges……………………………………0<br>Taking tablets daily is a challenge .... 1<br>Tablets improve health conditions slowly.......... 2<br>Tablets cause constipation and dark stools ...... 3<br>Tablets cause abdominal pain and vomiting or retching .... 4<br>Tablets are difficult for my body to absorb........ 5<br>I feel that tablets don't work very well .... 6<br>Other (specify) .... 98<br>Don't know……………………………………99	
6	Do you prefer oral iron tablets or IV iron?	Oral iron tablets .... 1<br>IV iron 2	
7	Why do you prefer _________ (INSERT RESPONSE FROM Q6)?	Open response	

Section B. IV Iron Experience

Now, I will ask you some questions about your experiences with receiving IV iron during your current visit to the hospital.

NO.	QUESTION	RESPONSE CODE	SKIP
1	Why did you decide to participate in the PRIORITY trial?	Open response	

NO.	QUESTION	RESPONSE CODE	SKIP
2	Who was involved in the decision-making process for your participation in the PRIORITY trial?<br><br>DO NOT READ RESPONSES. MULTIPLE RESPONSES POSSIBLE.	Myself .... 1<br>Spouse .... 2<br>Mother/ .... 3<br>Mother-in-law.. 4<br>Father……………………………………………..5<br>Father-in-law.……………………….............6<br>Health care provider…………………………….7<br>Other (SPECIFY):________...........................98	
	**Perceived benefits**		
3	You were randomly assigned to the IV iron study arm of the PRIORITY trial. What do you think are the benefits of getting IV iron?<br><br>DO NOT READ RESPONSES. MULTIPLE RESPONSES POSSIBLE.	No benefits…………………………………….0<br>It would improve my health .... 1<br>It would improve my baby's health.................... 2<br>It would make me stronger and give me more energy... 3<br>IV iron works better than oral iron .... 4<br>Only one dose is needed to get rid of anemia .. 5<br>IV iron has less side effects than oral iron .... 6<br>It would make me produce more breastmilk ..... 7<br>It would make it easier for me to take care of my baby.. 8<br>Other (SPECIFY): __________ .... 98<br>Don't know…………………………………….99	
	**Perceived severity**		
4	How did you feel after receiving IV iron?<br><br>DO NOT READ RESPONSES. MULTIPLE RESPONSES POSSIBLE.	Nothing happened……………………………0<br>I had bloating or swelling on one or more parts of the body.. 1<br>I felt dizzy or faint.. 2<br>I had nausea and cramps .... 3<br>I had problems with breathing.......................... 4<br>I had skin problems including rashes .... 5<br>Other (SPECIFY):________..........................98	
5	Did you experience any side effects or changes to your health after receiving the IV iron?	Yes .... 1<br>No 2	 → **B. Q8**
6	Did you tell your doctor, nurse, or the PRIORITY trial team about the side effect(s)?	Yes .... 1<br>No 2	
7	Did the doctor, nurse or PRIORITY trial team help you with this side effect or these side effects?	Yes .... 1<br>No 2	
8	Do you think there might be long-term side effects or changes from IV iron?	Yes .... 1<br>No .... 2<br>Don't know...3	 → **B. Q10**<br>→ **B. Q10**
9	What are the long-term side effects or changes?	Open response	
	**Perceived feasibility**		
10	What was your feeling regarding the process of IV iron infusion?	Very difficult .... 1<br>Somewhat difficult .... 2<br>Neither difficult, nor easy .... 3<br>Somewhat easy .... 4<br>Very easy... 5	

NO.	QUESTION	RESPONSE CODE	SKIP
11	What concerns did you have about receiving IV iron before you received it?<br><br>DO NOT READ RESPONSES. MULTIPLE RESPONSES POSSIBLE.	I had no concerns .... 1<br>I was afraid of having an infusion/needles .... 2<br>I was not sure it would work .... 3<br>I was afraid of side effects .... 4<br>Other (SPECIFY) 98	→ B. Q13
12	Do you still have those concerns?	Yes .... 1<br>No 2	
	**Acceptability of intervention (AIM, Proctor's)**		
13	Receiving IV iron met my expectations	Completely disagree .... 1<br>Disagree .... 2<br>Neither agree nor disagree .... 3<br>Agree .... 4<br>Completely agree 5	
14	Compared to oral iron, receiving IV iron was appealing to me	Completely disagree .... 1<br>Disagree .... 2<br>Neither agree nor disagree .... 3<br>Agree .... 4<br>Completely agree 5	
15	Receiving IV iron was better than I expected	Completely disagree .... 1<br>Disagree .... 2<br>Neither agree nor disagree .... 3<br>Agree .... 4<br>Completely agree 5	
16	I would agree to receive IV iron if needed again	Completely disagree .... 1<br>Disagree .... 2<br>Neither agree nor disagree .... 3<br>Agree .... 4<br>Completely agree 5	
	**Perceived barriers**		
17	What barriers did you face in receiving IV iron?<br><br>DO NOT READ RESPONSES. MULTIPLE RESPONSES POSSIBLE.	No barriers .... 1<br>I was concerned about the safety of drug .... 2<br>I had financial constraints for staying in the hospital .... 3<br>My family did not want me to receive IV iron .... 4<br>My family was not available to discuss receiving IV iron .... 5<br>Other (SPECIFY) 98	→ B. Q19
18	What can be done to address those barriers?	Open response	
	**Cost**		
19	Would you be willing to pay for IV iron treatment "out of pocket" if you needed IV iron in the future?	Yes .... 1<br>No 2	→ B. Q21
20	How much would you be willing to pay?	________ Rupees	→ B. Q22
21	Why not?	Open response	
	**Knowledge and beliefs**		
22	Do you think IV iron will make your anemia or symptoms of anemia better?	Yes .... 1<br>No 2	

NO.	QUESTION	RESPONSE CODE	SKIP
23	How likely are you to recommend IV iron to a family member or friend?<br><br>READ RESPONSES TO PARTICIPANT	Very unlikely .... 1<br>Somewhat unlikely .... 2<br>Neutral .... 3<br>Somewhat likely .... 4<br>Very likely 5	
	**Experiences with the PRIORITY trial team**		
24	Did the PRIORITY trial team answer any questions you had about the trial and about receiving IV iron?	Yes .... 1<br>No 2	
25	How comfortable or uncomfortable did you feel reaching out to the PRIORITY trial team?	Very uncomfortable .... 1<br>Somewhat uncomfortable .... 2<br>Neutral .... 3<br>Somewhat comfortable .... 4<br>Very comfortable 5	
26	How knowledgeable or lacking in knowledge was the PRIORITY trial team, in your opinion?	Very lacking in knowledge .... 1<br>Somewhat lacking in knowledge .... 2<br>Neutral .... 3<br>Somewhat knowledgeable .... 4<br>Very knowledgeable 5	
27	How skilled or lacking in skill was the PRIORITY trial team, in your opinion?	Very lacking in skill .... 1<br>Somewhat lacking in skill .... 2<br>Neutral .... 3<br>Somewhat skilled .... 4<br>Very skilled 5	
28	How kindly or unkindly did the PRIORITY trial team treat you?	Very unkindly .... 1<br>Somewhat unkindly .... 2<br>Neutral .... 3<br>Somewhat kindly .... 4<br>Very kindly 5	

Proctor's measures Psychometric assessment of three newly developed implementation outcome measures | Implementation Science | Full Text (biomedcentral.com)

Section C. Form Completion

1	Interviewer ID	_____________	
2	Date of interview	___/___/___ (month, day, year)	

This form should be completed by the *study staff* for all health workers (e.g., staff nurses) involved in the PRIORITY trial. Complete before ending the survey.

Section A. Introductions/Background

First, I will ask some background questions about your role.

0. Please select the site location.
    1. Jinnah Postgraduate Medical Center (JPMC) (Pakistan)
    2. General Hospital, Bailhongal (Belagavi, India)
    3. General Hospital, Hukkeri (Belagavi, India)
    4. General Hospital, Gokak (Belagavi, India)

1. What is your job title?
2. How many years have you worked at this job? [_] [_] . [_] years (use decimals for partial years)
3. What is your role in the PRIORITY trial?

Section B. IV Iron Implementation

In this questionnaire, when I ask questions about women, I am referring to postpartum women with moderate anemia. Questions about IV iron refer to single dose FCM IV iron.

CFIR Domain and Construct	Survey Question – referenced from CFIR website tools Tools – The Consolidated Framework for Implementation Research (cfirguide.org)
Intervention Characteristics: Evidence strength and quality	1. What kind of information is available to you related to giving FCM IV iron to postpartum women with anemia? MULTIPLE RESPONSES POSSIBLE. DO NOT READ RESPONSES.<br>1. Published literature<br>2. Co-worker experience<br>3. Practice guidelines<br>4. Patient experience<br>5. PRIORITY trial training<br>6. Doctor's advice<br>98. Other; please specify ____________<br>99. Don't know<br>2. How does this knowledge and information affect your confidence about providing FCM IV iron?<br>1. I am more confident providing IV iron.<br>2. I am less confident providing IV iron.

	3. I am neutral providing IV iron.
Intervention Characteristics: Relative advantage	3. Do you think that FCM IV iron or oral iron is better?<br>1. Yes, oral iron is a better treatment than IV iron<br>2. Yes, IV iron is a better treatment than oral iron<br>3. No, one treatment is not better than the other<br>4. What advantages does FCM IV iron have compared to oral iron? MULTIPLE RESPONSES POSSIBLE. DO NOT READ RESPONSES.<br>1. No advantages<br>2. Effective for most patients<br>3. More effective than oral iron<br>4. Patient compliance is assured<br>5. Patient has one visit<br>6. Ability to administer large dose in single infusion<br>7. No gastrointestinal side effects<br>98. Other; please specify _________________<br>99. Don't know<br>5. What disadvantages does FCM IV iron have compared to oral iron? MULTIPLE RESPONSES POSSIBLE. DO NOT READ RESPONSES.<br>1. No disadvantages<br>2. Adverse events<br>3. High cost<br>4. Requires trained and skilled staff<br>5. Requires equipment<br>6. Requires time to administer<br>7. Requires monitoring IV infusion<br>98. Other; please specify ___________________<br>99. Don't know
Intervention Characteristics: Complexity	6. What are the challenges in providing FCM IV iron to postpartum women with moderate anemia? MULTIPLE RESPONSES POSSIBLE. DO NOT READ RESPONSES.<br>1. No challenges<br>2. Length of time to administer IV iron<br>3. Number of steps to follow<br>4. Length of time to train workers to administer IV iron<br>5. Not enough trained or skilled workers to administer IV iron<br>6. Woman needs to be monitored constantly

	7. Ability to manage adverse events during or after infusion<br>8. Availability of emergency drugs for adverse events<br>98. Other; please specify __________________<br>99. Don't know<br>7. Providing FCM IV iron is more difficult for health workers than giving oral iron to postpartum women.<br>1. Strongly disagree<br>2. Disagree<br>3. Undecided<br>4. Agree<br>5. Strongly Agree
Outer Setting: Patient needs and resources	8. Based on your experience, what are the needs and preferences of postpartum women related to anemia treatment? DO NOT READ RESPONSES.<br>1. They prefer IV iron<br>2. They prefer oral iron<br>3. They prefer both IV iron and oral iron<br>4. They prefer nutrition counseling<br>98. Other, specify<br>99. Don't know<br>9. Before working with patients in the PRIORITY trial, how aware were you of the needs and preferences of postpartum women with moderate anemia?<br>1. Not aware at all<br>2. Slightly aware<br>3. Somewhat aware<br>4. Moderately aware<br>5. Extremely aware<br>10. In your opinion, were the needs and preferences of postpartum women served by your hospital considered before implementing this trial?<br>1. Yes, needs and preferences were considered<br>2. No, needs and preferences not considered at all<br>11. How well do you think the FCM IV iron used in this trial meets the needs of postpartum women with moderate anemia?<br>1. Not well at all<br>2. Slightly well<br>3. Somewhat well<br>4. Moderately well<br>5. Extremely well<br>12. What barriers do postpartum women face to receiving IV iron? MULTIPLE RESPONSES POSSIBLE. DO NOT READ RESPONSES.

	1. No barriers<br>2. Fear of injection<br>3. Cost<br>4. Think it is ineffective<br>5. Concern of side effects to self<br>6. Concern of side effects to child<br>98. Other; please specify ____________________<br>99. Don't know
Inner Setting: Implementation climate<br><br>Relative priority	13. How much of a priority is FCM IV iron for postpartum women compared to other health services?<br>1. Not a priority<br>2. Low priority<br>3. Medium priority<br>4. High priority<br>5. Essential<br>14. How easy or difficult is it to administer FCM IV iron compared to providing other services or completing your other responsibilities?<br>1. Very difficult<br>2. Difficult<br>3. Neutral<br>4. Easy<br>5. Very easy
Inner Setting: Readiness for implementation	15. How open are you to providing FCM IV iron to postpartum women after the PRIORITY trial ends?<br>1. Not at all<br>2. Slightly<br>3. Somewhat<br>4. Moderately<br>5. Extremely<br>16. Does the hospital have sufficient resources (such as*, funds, space, staff training, staff time, etc*.) to implement and administer FCM IV iron after the trial ends?<br>1. Yes<br>2. No<br>17. After the PRIORITY trial ends, will procuring necessary resources be a barrier to providing FCM IV iron to women postpartum?<br>1. Not a barrier<br>2. Somewhat of a barrier

	3. Moderate barrier<br>4. Extreme barrier
Individual Characteristics: Knowledge and beliefs about the intervention	18. How knowledgeable are you about FCM IV iron implementation?<br>1. Not at all knowledgeable<br>2. Slightly knowledgeable<br>3. Somewhat knowledgeable<br>4. Moderately knowledgeable<br>5. Extremely knowledgeable<br>99. Don't know
Individual Characteristics: Self-Efficacy	19. How confident are you that you can successfully administer FCM IV iron to postpartum women?<br>1. Not confident at all<br>2. Slightly confident<br>3. Somewhat confident<br>4. Fairly confident<br>5. Completely confident

Proctor's measures Psychometric assessment of three newly developed implementation outcome measures | Implementation Science | Full Text (biomedcentral.com)

**Feasibility of Intervention Measure (FIM)**	1. Completely disagree	2. Disagree	3. Neither agree nor disagree	4. Agree	5. Completely agree
20. Providing FCM IV iron to postpartum women seems implementable.	☐	☐	☐	☐	☐
21. Providing FCM IV iron to postpartum women seems easy.	☐	☐	☐	☐	☐

**Acceptability of Intervention Measure (AIM)**	1. Completely disagree	2. Disagree	3. Neither agree nor disagree	4. Agree	5. Completely agree
22. Providing FCM IV iron to postpartum women is acceptable to me.	☐	☐	☐	☐	☐
23. I have no objection to providing FCM IV iron to postpartum women.	☐	☐	☐	☐	☐

Section C. Form Completion

1	Interviewer ID	_____________
2	Date of interview	___/___/___ (month, day, year)

This form should be completed by the *study staff* for hospital administrators or support staff involved in the PRIORITY trial. Complete before ending the survey.

Section A. Introductions/Background

First, I will ask some background questions about your role.

0. Please select the site location.
    a. Jinnah Postgraduate Medical Center (JPMC) (Pakistan)
    b. General Hospital, Bailhongal (Belagavi, India)
    c. General Hospital, Hukkeri (Belagavi, India)
    d. General Hospital, Gokak (Belagavi, India)

1. What is your job title?
2. How many years have you worked in this job? [_] [_] . [_] years (use decimals for partial years)
3. What is your role in the PRIORITY trial?

Section B. IV Iron Implementation

In this questionnaire, when I ask questions about women, I am referring to postpartum women with moderate anemia. Questions about IV iron refer to single dose FCM IV iron.

CFIR Domain and Construct	Survey Question – referenced from CFIR website tools Tools – The Consolidated Framework for Implementation Research (cfirguide.org)
Intervention Characteristics: Evidence strength and quality	1. What kind of information is available to you related to giving FCM IV iron to postpartum women with anemia? MULTIPLE RESPONSES POSSIBLE. DO NOT READ RESPONSES.<br>1. Published literature<br>2. Co-worker experience<br>3. Practice guidelines<br>4. Patient experience<br>5. PRIORITY trial training<br>6. Doctor's advice<br>98. Other; please specify _____________<br>99. Don't know<br>2. How does this knowledge and information affect your confidence about giving women FCM IV iron?<br>1. I am more confident providing IV iron.<br>2. I am less confident providing IV iron.<br>3. I am neutral about providing IV iron.

Intervention Characteristics: Relative advantage	3. Do you think that FCM IV iron or oral iron is better?<br>1. Yes, oral iron is better than IV iron<br>2. Yes, IV iron is better treatment than oral iron<br>3. No, one treatment is not better than the other<br>4. What advantages does FCM IV iron intervention have compared to oral iron? MULTIPLE RESPONSES POSSIBLE. DO NOT READ RESPONSES.<br>1. No advantages<br>2. Effective for most patients<br>3. More effective than oral iron<br>4. Patient compliance is assured<br>5. Patient has one visit<br>6. Ability to administer large dose in single infusion<br>7. No gastrointestinal side effects<br>98. Other; please specify ________________<br>99. Don't know<br>5. What disadvantages does FCM IV iron intervention have compared to oral iron? MULTIPLE RESPONSES POSSIBLE. DO NOT READ RESPONSES.<br>1. No disadvantages<br>2. Adverse reactions<br>3. High cost<br>4. Requires trained and skilled staff<br>5. Requires equipment<br>6. Requires time to administer<br>7. Requires monitoring IV infusion<br>98. Other; please specify __________________<br>99. Don't know
Intervention Characteristics: Complexity	6. What are the challenges in providing IV iron to postpartum women with moderate anemia? MULTIPLE RESPONSES POSSIBLE. DO NOT READ RESPONSES.<br>1. No challenges<br>2. Length of time to administer IV iron<br>3. Number of steps to follow<br>4. Length of time to train workers to administer IV iron<br>5. Not enough trained or skilled workers to administer IV iron<br>6. Woman needs to be monitored constantly<br>7. Ability to manage adverse events during or after infusion<br>8. Availability of emergency drugs for adverse events<br>98. Other; please specify _________________<br>99. Don't know

	1.
Outer Setting: Patient needs and resources	7. In your opinion, what are the needs and preferences of postpartum women with moderate anemia in terms of anemia treatment? DO NOT READ RESPONSES.<br>1. They prefer IV iron<br>2. They prefer oral iron<br>3. They prefer both IV iron and oral iron<br>4. They prefer nutrition counseling<br>98. Other, specify<br>99. Don't know<br><br>8. How aware are hospital staff of the needs and preferences of postpartum women with moderate anemia?<br>1. Not aware at all<br>2. Slightly aware<br>3. Somewhat aware<br>4. Moderately aware<br>5. Extremely aware<br>9. Were the needs and preferences of postpartum women served by your hospital considered before implementing this trial?<br>1. Yes, needs and preferences were considered<br>2. No, Needs and preferences not considered at all<br>10. How well do you think the FCM IV iron used in this trial will meet the needs of the postpartum women with moderate anemia?<br>1. Not well at all<br>2. Slightly well<br>3. Somewhat well<br>4. Moderately well<br>5. Extremely well<br>11. What barriers do postpartum women face to receiving FCM IV iron? MULTIPLE RESPONSES POSSIBLE. DO NOT READ RESPONSES.<br>1. No barriers<br>2. Fear of injection<br>3. Cost<br>4. Think it is ineffective<br>5. Concern of side effects to self<br>6. Concern of side effects to child<br>98. Other; please specify ______________________<br>99. Don't know

Outer Setting: External policies	12. What factors influenced the decision to implement the PRIORITY trial in this hospital? MULTIPLE RESPONSES POSSIBLE. DO NOT READ RESPONSES.<br>1. Nothing influenced the decision<br>2. Hospital was approached to participate in the trial<br>3. Local policies<br>4. State policies<br>5. National policies<br>6. Hospital clinical performance measures<br>7. Financial incentive<br>98. Other; specify _______________<br>99. Don't know
Inner Setting: Implementation climate<br><br>Relative priority	13. How much of a priority is FCM IV iron for postpartum women compared to other health services?<br>1. Not a priority<br>2. Low priority<br>3. Medium priority<br>4. High priority<br>5. Essential<br>14. How easy or difficult is it to administer FCM IV iron postpartum compared to providing other priority services?<br>1. Very difficult<br>2. Difficult<br>3. Neutral<br>4. Easy<br>5. Very easy
Inner Setting: Readiness for implementation	15. How open are hospital staff to providing FCM IV iron to postpartum women after the PRIORITY trial ends?<br>1. Not at all receptive<br>2. Slightly receptive<br>3. Somewhat receptive<br>4. Moderately receptive<br>5. Extremely receptive<br>16. Does the hospital have sufficient resources (such as*, funds, space, staff training, staff time, etc*.) to implement and administer FCM IV iron after the trial ends?<br>1. Yes<br>2. No<br>17. After the PRIORITY trial ends, will procuring necessary resources be a barrier to providing FCM IV iron to women postpartum?<br>1. Not a barrier<br>2. Somewhat of a barrier

	3. Moderate barrier<br>4. Extreme barrier<br>98. Other, please specify _________________
Individual Characteristics: Knowledge and beliefs about the intervention	18. How knowledgeable are you about FCM IV iron implementation?<br>1. Not at all knowledgeable<br>2. Slightly knowledgeable<br>3. Somewhat knowledgeable<br>4. Moderately knowledgeable<br>5. Extremely knowledgeable<br>99. Don't know
Individual Characteristics: Self-Efficacy	19. How confident are you that the staff of this hospital can successfully implement FCM IV iron for postpartum women?<br>1. Not confident at all<br>2. Slightly confident<br>3. Somewhat confident<br>4. Fairly confident<br>5. Completely confident

Proctor's measures Psychometric assessment of three newly developed implementation outcome measures | Implementation Science | Full Text (biomedcentral.com)

**Feasibility of Intervention Measure (FIM)**	1. Completely disagree	2. Disagree	3. Neither agree nor disagree	4. Agree	5. Completely agree
20. Providing FCM IV iron to postpartum women seems implementable.	□	□	□	□	□
21. Providing FCM IV iron to postpartum women seems easy.	□	□	□	□	□

**Acceptability of Intervention Measure (AIM)**	1. Completely disagree	2. Disagree	3. Neither agree nor disagree	4. Agree	5. Completely agree
22. Providing FCM IV iron to postpartum women is acceptable to me.	□	□	□	□	□
23. I have no objection to providing FCM IV iron to postpartum women.	□	□	□	□	□

Section C. Form Completion

1	Interviewer ID	______________

2	Date of interview	___/___/___ (month, day, year)

This form should be completed by the *study staff* for women who refuse to enroll in the PRIORITY trial using information from the interview. Complete before ending the exit survey for women who refused to enroll in the trial.

Section A. Introduction/Background

First, I will ask some questions about you and your experience with anemia.

NO.	QUESTION	RESPONSE CODE	SKIP
0	Please select the site location.	Jinnah Postgraduate Medical Center (JPMC) (Pakistan)……………………………………….1<br>General Hospital, Bailhongal (Belagavi, India) …………………………………………………….2<br>General Hospital, Hukkeri (Belagavi, India) …………………………………………………….3<br>General Hospital, Gokak (Belagavi, India) …………………………………………………….4	
1	How old are you?	[__][__] AGE IN YEARS	
2	Has a doctor, nurse, or other health worker ever told you that you have anemia during this pregnancy?	Yes .... 1<br>No 2	 **→ B. Q1**
3	Did you take iron tablets to treat anemia?	Yes .... 1<br>No 2	 **→ B. Q1**
4	How many weeks did you take the iron tablets for anemia?	[__][__] WEEKS OF IRON TABLET USE FOR ANEMIA	

Section B. IV Iron Experience

Now, I will ask you some questions about your thoughts about IV iron during your current visit to the hospital.

NO.	QUESTION	RESPONSE CODE	SKIP
	**Willingness to participate**		
1	Why did you decide **not** to participate in the PRIORITY trial?<br><br>DO NOT READ RESPONSES. MULTIPLE RESPONSES POSSIBLE.	I prefer oral iron to IV iron................................ 1<br>I did not think it was effective ....2<br>I did not think I needed it ....3<br>IV iron is too difficult to take……………………………4<br>A doctor/nurse did not recommend it………………….5<br>I am afraid of needles/IV..……………………………....6<br>I don't want to participate in research………………..7<br>I did not know which treatment I would receive……...8<br>I wasn't given permission to participate……………..9<br>I don't want to spend extra time in the hospital…….10<br>A family member did not recommend it (Specify)______...11<br>A friend did not recommend it (Specify) ________..12<br>Other (SPECIFY): __________ ……………………..98	
2	Who was involved in the decision-making process when you decided not to participate in the PRIORITY trial?	Myself……………………………………………….1<br>Spouse……………………………………………..2<br>Mother/Mother-in-law……………………………3<br>Father/Father-in-law……………………………..4<br>Other (SPECIFY):________...........................98	
	**Perceived benefits**		
3	What do you think are the benefits of getting IV iron, if any?	It would improve my health................................1<br>It would improve my baby's health 2	

NO.	QUESTION	RESPONSE CODE	SKIP
	DO NOT READ RESPONSES. MULTIPLE RESPONSES POSSIBLE.	It would make me stronger and give me more energy ....3<br>IV iron works better than oral iron ....4<br>Only one dose is needed to get rid of anemia...5<br>IV iron has less side effects than oral iron.........6<br>It would make me produce more breastmilk......7<br>It would make it easier for me to take care of my baby ....8<br>Other (SPECIFY): __________ 98	
	**Perceived severity**		
4	Do you think there would be negative side effects or changes to your health were you to receive IV iron?	Yes .... 1<br>No 2	→ **B. Q6**
5	If yes, what side effects do you think will occur?<br><br>DO NOT READ RESPONSES. MULTIPLE RESPONSES POSSIBLE.	Shivering……………………………………………1<br>Fever…………………………………………………2<br>Pain in Abdomen …………………………………3<br>Fainting ……………………………………………4<br>Palpitation …………………………………………5<br>Sweating ……………………………………………6<br>Other (Specify) ________________...............98	
6	Do you think there might be long-term negative side effects or changes because of getting IV iron?	Yes .... 1<br>No 2	→ **B. Q8**
7	What types of long-term side effects were you worried about?	Open response	
	**Perceived feasibility**		
8	How easy or difficult do you think is it to receive IV iron?	Very difficult...1<br>Somewhat difficult ....2<br>Neither easy nor difficult...................................3<br>Somewhat easy..4<br>Very easy 5	
	**Acceptability of intervention (AIM, Proctor's)**		
9	Compared to oral iron, receiving IV iron is appealing to me	Completely disagree ....1<br>Disagree..2<br>Neither agree nor disagree...............................3<br>Agree ....4<br>Completely agree 5	
10	I would agree to receive IV iron if needed	Completely disagree ....1<br>Disagree..2<br>Neither agree nor disagree...............................3<br>Agree ....4<br>Completely agree 5	
	**Perceived barriers**		
11	If you wanted to take IV iron, what barriers, if any, would you face?<br><br>DO NOT READ RESPONSES. MULTIPLE RESPONSES POSSIBLE.	Allergic or infusion reactions………………………1<br>Requires continuous monitoring by providers…..2<br>Higher cost ……………………………………………3<br>Fear of infusions………………………………………4<br>Lack of trained staff…………………………………5<br>My family doesn't want/allow me to have it…….6<br>Other (SPECIFY)…………………………………98	
12	What could be done to address those barriers?	Open response	

NO.	QUESTION	RESPONSE CODE	SKIP
	**Cost**		
13	Would you be willing to pay for IV iron treatment "out of pocket" if you needed it in the future?	Yes .... 1<br>No 2	→ **B. Q15**
14	How much would you be willing to pay?	________ Rupees	→ **B. Q16**
15	Why not?	Open response	
	**Knowledge and beliefs**		
16	Do you think IV iron would make your anemia or symptoms of anemia better?	Yes .... 1<br>No 2	
	**Experiences with PRIORITY trial team**		
17	Did the PRIORITY trial team explain what would happen to you if you decided to participate in the trial, including any benefits?	Yes .... 1<br>No 2	
18	Did the PRIORITY trial team explain what would happen to you if you decided to participate in the trial, including any possible side effects?	Yes .... 1<br>No 2	
19	Did the PRIORITY trial team answer any questions you had about the trial?	Yes .... 1<br>No 2	

Proctor's measures Psychometric assessment of three newly developed implementation outcome measures | Implementation Science | Full Text (biomedcentral.com)

Section C. Form Completion

1	Interviewer ID	____________
2	Date of interview	___/___/___ (month, day, year)

This guide should be used by the *study staff* for women enrolled in the PRIORITY trial and who are receiving IV iron.

You are being asked to be in a research study to learn about the best ways to treat women who have moderate anemia immediately after giving birth and to understand the facilitators, barriers, and cost of implementing **single dose** IV iron. We would like to spend about 1 hour to ask you some questions about your experience with IV iron.

Participant ID (use same ID as for PRIORITY trial)	
Interviewer's name/ID	
Notetaker's name/ ID	
Name of Health Facility	
Date of Interview	
Starting time of Interview	
Ending time of Interview	

Introductions/Background/Demographics

1. How old are you? _______years
2. How many years of schooling have you completed? ________years
3. How many children do you have?
4. What region or locality are you from?

Experience with IV Iron – what was women's experience with IV iron?

5. Please tell us what you remember about receiving IV iron.
6. [Perceived barriers/complexity] What barriers did you face in receiving IV iron?
    a. Were you concerned about staying in the hospital longer? Tell me more about that.
    b. Were you concerned about your health? Tell me more about that.
    c. What can be done to address these barriers?
7. [Willingness to participate] Tell us about your decision to participate in the PRIORITY trial. What led you to join the trial?
    a. Did someone recommend it to you? If so, who recommended it?
8. [Perceived severity] How serious do you think anemia is after delivery? Please explain.
9. [Perceived benefits] How would you describe the benefits of receiving IV iron? Now and in the future?
10. [Perceived ease/feasibility] How easy or difficult was it to receive IV iron?
    a. What made it easy/difficult?
    b. What could have been done to make the experience better?
11. [Perceived barriers/complexity] What if any, were your concerns about receiving IV iron before you started the trial? Do you still have those concerns? Tell me more about that.
12. [Cost] Would you or your family be willing to pay for IV iron treatment if you needed it again in the future? Please explain.
13. Would you recommend IV iron to a family member or friend who has anemia? Please explain.
14. Have you previously received iron tablets for anemia?
    a. How do you compare your experiences receiving iron tablets with receiving IV iron?
    b. What did you like about tablets versus IV iron?
    c. What did you dislike about tablets versus IV iron?

Experience with PRIORITY team – The PRIORITY trial team refers to the doctors, nurses, or health care professionals involved in the PRIORITY trial.

15. Tell us about your experience with the PRIORITY trial team.
16. What did the PRIORITY trial team explain to you about the trial?
17. How well did the PRIORITY trial team answer any questions you had about the trial? Tell me more about that.
18. How comfortable did you feel about reaching out to the PRIORITY trial team if you experienced any side effects? Tell me more about that.
19. In your opinion, how confident was the PRIORITY trial team about providing IV iron to you? Tell me more about that.
20. In your opinion, how skilled was the PRIORITY trial team in giving you the IV iron? Tell me more about that.
21. In your opinion, how kindly were you treated by the PRIORITY trial team?
    a. What could the PRIORITY trial team have done to make you feel more comfortable? Tell me more about that.
22. Is there anything else you would like to add?

This guide should be completed by the *study staff* for family members of women who are enrolled in the PRIORITY trial and are receiving IV iron.

You are being asked to be in a research study to learn about the best ways to treat women who have moderate anemia immediately after giving birth and to understand the facilitators, barriers, and cost of implementing IV iron. We would like to spend about 1 hour to ask you some questions about your experience with your family member receiving **single dose** IV iron.

Participant ID	
Type of Participant (husband, mother, mother-in-law)	
Age of participant	
Interviewer's name/ID	
Notetaker's name/ID	
Name of Health Facility	
Date of Interview	
Starting time of Interview	
Ending time of Interview	

Experience with Anemia

1. Tell us about your experience with anemia.
    a. Do you know anyone who has or had anemia postpartum, other than your [wife/daughter/daughter-in-law] who is enrolled in the PRIORITY trial? How would you describe her health at that time? What did you observe?
    b. When you learned your [wife/daughter/daughter-in-law] had anemia, what did you think?

Experience with IV Iron – what was women's experience with IV iron?

2. [Willingness to participate] Tell us about your [wife/daughter/daughter-in-law]'s experiences with IV iron.
    a. Did you support her decision to participate in the trial? What were your reasons for supporting her decision or not?
    b. Did she tell you why she decided to participate in the trial? What did she share?
    c. Did someone recommend it to her? Who was that person and why did he or she recommend it?
3. [Perceived severity] When you learned that your [wife/daughter/daughter-in-law] was going to receive IV iron, what were your concerns or questions?
4. [Perceived severity or side effects] Did you discuss the IV iron infusion with your [wife/daughter/daughter-in-law] before she had it? If so, what concerns did she share with you? What concerns does she still have?
5. [Perceived benefits] What do you think are the benefits of getting IV iron? Have you noticed any of these benefits in your [wife/daughter/daughter-in-law] since she got the IV iron? Which ones?
6. [Perceived ease/feasibility] Were you involved in helping before, during or after the IV infusion? What did you think about the process?
7. [Perceived barriers/complexity] What are some challenges your [wife/daughter/daughter-in-law] shared with you related to receiving IV iron? What can be done to address these challenges?
8. [Cost] Would you be willing to pay for IV iron treatment for your [wife/daughter/daughter-in-law] if she needed it again in the future? Please explain your reasons.
9. Would you recommend IV iron to a family member or friend who has anemia? Tell me more about this.

Experience with PRIORITY trial team – The PRIORITY trial team refers to the doctors, nurses, or health care professionals involved in the IV iron trial.

10. Were you present when the PRIORITY trial team was talking to your [wife/daughter/daughter-in-law]? Or did the PRIORITY trial team talk to you separately? *If the PRIORITY trialteam talked to the husband/mother/mother-in-law, continue to the next questions.*

11. What did the PRIORITY trial team explain about the trial?
12. How well did the PRIORITY trial team answer any questions you had about the trial? Tell me more about that.
13. How comfortable did you feel about reaching out to the PRIORITY trial team if your [wife/daughter/daughter-in-law] experienced any side effects? Tell me more about that.
14. In your opinion, how confident was the PRIORITY trial team about providing IV iron? Tell me more about that.
15. In your opinion, how skilled was the PRIORITY trial team in giving your [wife/daughter/daughter-in-law] the IV iron? Tell me more about that.
16. In your opinion, how kindly was your [wife/daughter/daughter-in-law] treated by the PRIORITY trial team? Tell me more about that.
17. What could the PRIORITY trial team have done to make your [wife/daughter/daughter-in-law] feel more comfortable? What could the PRIORITY trial team have done to make you feel more comfortable? Tell me more about that.
18. Do you have anything else you would like to add?

This guide should be completed by the *study staff* for health workers and administrators who are screened for participation in the implementation research study using information from this in-depth interview.

You are being asked to be in a research study to learn about the best ways to treat women who have moderate anemia immediately after giving birth and to understand the facilitators, barriers, and cost of implementing IV iron. We would like to spend about 1 hour to ask you some questions about your experience with administering IV iron.

Participant ID	
Interviewer's name/ID	
Notetaker's name/ID	
Name of Health Facility	
Date of Interview	
Starting time of Interview	
Ending time of Interview	

Introductions/Background

First, I will ask some background questions about your role.

1. What is your job title?
2. How many years have you worked at this job?
3. What is your role in the PRIORITY trial?

I would now like to ask you questions about IV iron for postpartum women with moderate anemia. The questions refer specifically to FCM IV iron, not to iron sucrose.

FOR BOTH HEALTH WORKERS AND HOSPITAL ADMINISTRATORS:

**CFIR Domain and Construct**	**IDI Question – referenced from CFIR website tools** [Tools – The Consolidated Framework for Implementation Research (cfirguide.org)]
Intervention Characteristics: Evidence strength and quality	1. From your perspective, what kind of information or evidence are you aware of related to giving a single dose of FCM IV iron to women postpartum? Probes: Have you heard of this information from practice guidelines? published literature? co-workers? other places?<br>2. Now that you are involved in research related to FCM IV iron, how would you explain the purpose of a single dose of IV iron to a patient?
Intervention Characteristics: Relative advantage	3. How does administering FCM IV iron compare to administering oral iron to postpartum women with moderate anemia?<br>4. What advantages does FCM IV iron have compared to oral iron?<br>5. What disadvantages does FCM IV iron have compared to oral iron?
Outer Setting: Patient needs and resources	6. What have postpartum women told you about their experiences with FCM IV iron? Probe for specific information or examples.<br>7. Aside from the health benefits of FCM IV iron, how else can IV iron be beneficial for women? Probe on improved access to services, reduced wait times, reduced follow up visits, reduced travel time and expense<br>8. What barriers do postpartum women face to receiving FCM IV iron?

	a. Probe on costs, family support, time needed, transportation issues<br>9. How can administering FCM IV iron be altered to address these barriers?
Inner Setting: Implementation climate<br><br>Relative priority	If FCM IV iron is proven effective for treating moderate anemia postpartum…<br>10. How important do you think it is to implement FCM IV iron for postpartum women compared to other priorities in this hospital?

FOR HEALTH WORKERS ONLY:

Intervention Characteristics: Complexity	11. How would you describe the process of providing FCM IV iron to a postpartum patient?<br>a. How long does it take to provide IV iron?<br>b. How many steps are involved to provide IV iron?<br>c. How difficult are the steps?
Implementation strategies	12. When working with patients in the PRIORITY trial, what do you do to make it easier for yourself and the patient to carry out the steps required to implement IV iron?<br>13. If you were talking to a colleague who had never given IV iron to postpartum women, what suggestions would you give them to make it easier? Probe on suggestions for working with patients and their families and for the technical aspects of providing IV iron.
Individual Characteristics: Knowledge and beliefs about the intervention	14. How do you personally feel about FCM IV iron being used for moderately anemic women postpartum?<br>a. Probe for feelings of anticipation, stress, enthusiasm
Individual Characteristics: Self-Efficacy	15. How has your confidence to give FCM IV iron changed since the beginning of the PRIORITY trial?

FOR HOSPITAL ADMINISTRATORS ONLY:

Outer Setting: External policies	16. What kind of factors would influence the hospital's decision to provide FCM IV iron after the PRIORITY trial ends?<br>17. What kind of financial or other incentives would influence the hospital's decision to implement FCM IV iron after the PRIORITY trial ends?<br>18. How will administering FCM IV iron affect payment or revenue for your hospital in the future?
Inner Setting: Readiness for implementation	19. How do you expect to procure necessary resources to implement FCM IV iron for postpartum women?<br>a. What resources do you already have?<br>b. What resources are needed?<br>c. Who will be involved in helping you get what is needed?<br>d. What challenges do you expect to encounter?<br>20. What other barriers for health workers and for the hospital are there to implementing IV iron?<br>21. How can these barriers be addressed?

Process: Formally appointed internal implementation leaders/champions	22. Has this hospital appointed an implementation leader or champion for FCM IV iron? Why or why not?<br>23. Which leaders or influential individuals at the hospital need to be on board for the implementation of FCM IV iron to be successful?

PRIORITY IV Iron Infusion Checklist

Instructions: Observers (which may include the IPI, country coordinator, or study coordinator) should complete this checklist form when supervising IV iron infusion activities as part of the PRIORITY trial. The form should be completed at least **once per month for each health care provider (HCP)** who is responsible for conducting IV infusion activities for PRIORITY. One form should be completed for a single infusion.

ID of Health Care Provider #1: _______________ ID of Health Care Provider #2: _______________

Observer ID: ______________________________ Date: |__|__|/|__|__|/|__|__|__|__| (dd/mm/yyyy)

STEP	COMPLETE? (Y/N/NA)	COMMENTS OR CORRECTIVE ACTIONS MADE BY THE OBSERVER. SPECIFY FOR WHICH HEALTH CARE PROVIDER.
**INSTRUCTIONS:** VISUALLY OBSERVE EACH ACTIVITY IN THE CHECKLIST BELOW. SELECT "YES", "NO", OR "N/A" TO INDICATE IF THE HEALTH CARE PROVIDERS HAVE COMPLETED THE ACTIVITY AS REQUIRED BY THE IV IRON INFUSION SOP. FOR ALL "NO" RESPONSES, THE OBSERVER MUST PROVIDE FURTHER DETAILS IN THE COMMENT FIELD, INCLUDING ANY CORRECTIVE ACTION PROVIDED. FOR "YES" AND "N/A" RESPONSES, COMMENTS ARE OPTIONAL.		
**A. Check the IV Infusion set and participant**		
1. The health care provider checked if there is any evidence of damage to the IV infusion set.	Y/ N	1a.
1b. If identified, the health care provider replaced the set and logged the issue.	Y/N/NA	
2. The health care provider inspected the IV iron vial for visible precipitation or particulate matter.	Y/N	2a.
2b. If identified, the health care provider replaced the vial and logged the issue.	Y/N/NA	2c.
3. The health care provider recorded all vital signs on PT03.	Y/N	3a.
3b. If there were irregularities in any of the vital signs, they were taken again to confirm whether they remained irregular.	Y/N/NA	3c.
3d. If they remained irregular, the health care provider used judgement regarding whether to delay or cancel the infusion.	Y/N/NA	3e.
4. The health care provider did not give any kind of pre-medication (e.g., anti-histaminic, steroids) before administration of the IV iron drug.	Y/N	4a.
5. The health care provider counseled the participant about the procedure and what to expect.	Y/N	5a.
5b. The health care provider answered any questions in a reassuring manner.	Y/N/NA	5c.

PRIORITY IV Iron Infusion Checklist

STEP	COMPLETE? (Y/N/NA)	COMMENTS OR CORRECTIVE ACTIONS MADE BY THE OBSERVER. <u>SPECIFY FOR WHICH HEALTH CARE PROVIDER.</u>
**INSTRUCTIONS:** VISUALLY OBSERVE EACH ACTIVITY IN THE CHECKLIST BELOW. SELECT "YES", "NO", OR "N/A" TO INDICATE IF THE HEALTH CARE PROVIDERS HAVE COMPLETED THE ACTIVITY AS REQUIRED BY THE IV IRON INFUSION SOP. FOR ALL "NO" RESPONSES, THE OBSERVER MUST PROVIDE FURTHER DETAILS IN THE COMMENT FIELD, INCLUDING ANY CORRECTIVE ACTION PROVIDED. FOR "YES" AND "N/A" RESPONSES, COMMENTS ARE OPTIONAL.		
**B. Administration of IV iron: Ferric Carboxymaltose (FCM)**		
1. The health care provider started the normal saline first.	Y/N	1a.
2. The health care provider then added the IV iron into normal saline bottle.	Y/N	2a.
3. The health care provider inverted (turned up and down to mix solution) the IV iron bag after iron was added to the infusion bag.	Y/N	3a.
4. The health care provider started the timer to monitor the infusion when the health care provider saw the drug (brown liquid) enter the cannula.	Y/N	4a.
5. The health care provider closely observed the participant for 5 minutes before increasing the infusion administration rate.	Y/N	5a.
6. The health care provider completed the infusion within 25 to 30 minutes.	Y/N	6a.
**C. Monitoring**		
1. Two health care providers were in the room with the participant to monitor the infusion at all times.	Y/N	1a.
2. If there was any evidence that the participant was not tolerating the infusion, the health care provider immediately paused the IV iron infusion but left the cannula in place in case emergency medications were required.	Y/N/NA	2a.
During the infusion (3 types of monitoring)		
3. **Visual observation of participant:** The health care provider visually observed the participant for any side effects such as headache, breathlessness, dizziness, hypertension, abdominal pain, nausea, or anaphylactic reactions during the drug administration.	Y/N	3a.
4. **Verbal communication with the participant:** The health care provider verbally assessed the participant by asking questions (e.g., "How are you feeling?").	Y/N	4a.

PRIORITY IV Iron Infusion Checklist

STEP	COMPLETE? (Y/N/NA)	COMMENTS OR CORRECTIVE ACTIONS MADE BY THE OBSERVER. SPECIFY FOR WHICH HEALTH CARE PROVIDER.
**INSTRUCTIONS:** VISUALLY OBSERVE EACH ACTIVITY IN THE CHECKLIST BELOW. SELECT "YES", "NO", OR "N/A" TO INDICATE IF THE HEALTH CARE PROVIDERS HAVE COMPLETED THE ACTIVITY AS REQUIRED BY THE IV IRON INFUSION SOP. FOR ALL "NO" RESPONSES, THE OBSERVER MUST PROVIDE FURTHER DETAILS IN THE COMMENT FIELD, INCLUDING ANY CORRECTIVE ACTION PROVIDED. FOR "YES" AND "N/A" RESPONSES, COMMENTS ARE OPTIONAL.		
5. **Visual observation of the IV line:** The health care provider observed the IV line to ensure the drip had a consistent flow and the line was intact throughout the procedure.	Y/N	5a.
After the Infusion		
6. The health care provider visually observed the participant for at least 30 minutes.	Y/N	6a.
7. The health care provider asked the participant how she was feeling.	Y/N	7a.
8. The health care provider kept the cannula in place until the 30-minute post-infusion monitoring period was completed in case emergency medications were required.	Y/N	8a.
9. If the participant was not comfortable after 30 minutes, the health care provider extended the monitoring period for another 30 minutes.	Y/N/NA	9a.
**D. Discharge**		
Before participant leaves the infusion room, did the health care provider complete the following discharge procedures?		
1. The health care provider showed the participant the contents of the folic acid box while reviewing the "Discharge Hand-out for IV Iron Arm".	Y/N	1a.
2. The health care provider asked the participant to remove one folic acid pill and take it.	Y/N	2a.
3. The health care provider watched the participant take pill (and ensured that water or other drink was available).	Y/N	3a.
4. The health care provider recorded pill consumption in drug accountability logbook (e.g., date, time, and blister strip #, etc.).	Y/N	4a.
5. After the participant took the pill, the health care provider returned the blister strip to the folic acid box and asked participant to take box with her.	Y/N	5a.
6. The health care provider reminded the participant that she should take another pill tomorrow.	Y/N	6a.
