# Supplementary material for: Using implementation research frameworks to identify facilitators and barriers to implementation of ferric carboxymaltose intravenous iron among moderately anemic postpartum women participating in a trial in India
Source: PLoS One. 2026 Oct 21;21(10):e0359787. doi: 10.1371/journal.pone.0359787 (PMC13686276; doi:10.1371/journal.pone.0359787)
Supplement: Supplementary file 2 [file pone.0359787.s001.docx]

**S1 Table:** Good Reporting of a Mixed Methods Study (GRAMMS) checklist

| **Item** | **Reported** |
| --- | --- |
| Describe the justification for using a mixed methods approach to the research question | Study design, page 5 |
| Describe the design in terms of the purpose, priority, and sequence of methods | Study design, page 5 |
| Describe each method in terms of sampling, data collection, and analysis | Study sites and participants, Data collection, Data analysis, pages 6-11 |
| Describe where integration has occurred, how it has occurred, and who has participated in it | Data analysis, page 11 |
| Describe any limitation of one method associated with the presence of the other method | Limitations were related to conducting the study within a clinical trial, not to the use of different methods. Limitations are described on page 37 |
| Describe any insights gained from mixing or integrating methods | Discussion, page 33-34 |

Reference: O'Cathain A, Murphy E, Nicholl J. The quality of mixed methods studies in health

services research. J Health Serv Res Policy. 2008;13(2):92-98.
