# Supplementary material for: Using implementation research frameworks to identify facilitators and barriers to implementation of ferric carboxymaltose intravenous iron among moderately anemic postpartum women participating in a trial in India
Source: PLoS One. 2026 Oct 21;21(10):e0359787. doi: 10.1371/journal.pone.0359787 (PMC13686276; doi:10.1371/journal.pone.0359787)
Supplement: Supplementary file 3 [file pone.0359787.s002.docx]

**S2 Table.** Resources Available in Government Hospitals During the PRIORITY Trial Compared with Indian Sub-district Hospitals Under Usual Conditions

|  | **Resources available during the trial** | **Resources in an Indian sub-district hospital under usual conditions** |
| --- | --- | --- |
| FCM supply | Yes | No  They must procure from the allocated budget, so they usually have iron sucrose. |
| 10ml syringe supply | Regular | Irregular |
| Protocol for FCM IV infusion | Yes | No |
| Staff trained on FCM infusion | Yes | No |
| Supervision of nurses who provide intravenous iron | Yes | Yes, but for iron sucrose, not FCM |
| Separate room for infusion | Yes | No |
| Emergency kit in case of a drug reaction, including hydrocortisone and methylprednisolone | Yes | No |
