# Supplementary material for: Using implementation research frameworks to identify facilitators and barriers to implementation of ferric carboxymaltose intravenous iron among moderately anemic postpartum women participating in a trial in India
Source: PLoS One. 2026 Oct 21;21(10):e0359787. doi: 10.1371/journal.pone.0359787 (PMC13686276; doi:10.1371/journal.pone.0359787)
Supplement: Supplementary file 4 [file pone.0359787.s004.docx]

**S4 Table.** Survey Findings from Health Workers and Hospital Administrators Related to CFIR Domains and Proctor’s Outcomes by Hospital

|  | **Hukkeri** | **Bailhongal** | **Gokak** |
| --- | --- | --- | --- |
| **CFIR Domains and Constructs** | **n** | **n** | **n** |
| **Intervention characteristics** | **N=5** | **N=5** | **N=6** |
| Evidence strength and quality |  |  |  |
| Sources of information about FCM IV iron for postpartum women with anemia |  |  |  |
| Published literature | 2 | 1 | 2 |
| Co-worker experience | 0 | 2 | 1 |
| Practice guidelines | 2 | 3 | 4 |
| Patient experience | 3 | 2 | 1 |
| PRIORITY trial training | 5 | 4 | 5 |
| Doctor's advice | 0 | 2 | 2 |
| Other | 0 | 1 | 1 |
| How this information affects your confidence about providing FCM IV iron |  |  |  |
| I am more confident providing IV iron | 4 | 3 | 6 |
| I am less confident providing IV iron | 1 | 1 | 0 |
| I am neutral providing IV iron | 0 | 1 | 0 |
| Relative advantage |  |  |  |
| Is FCM IV iron or oral iron better in your opinion |  |  |  |
| Yes, oral iron is a better treatment than IV iron | 1 | 0 | 1 |
| Yes, IV iron is a better treatment than oral iron | 4 | 5 | 4 |
| No, one treatment is not better than the other | 0 | 0 | 1 |
| Advantages of FCM IV iron compared to oral iron |  |  |  |
| Effective for most patients | 5 | 2 | 1 |
| More effective than oral iron | 4 | 3 | 2 |
| Patient compliance is assured | 4 | 4 | 6 |
| Patient has one visit | 5 | 2 | 3 |
| Ability to administer large dose in single infusion | 3 | 3 | 6 |
| Little or no side effects | 0 | 0 | 1 |
| Disadvantages of FCM IV iron compared to oral iron |  |  |  |
| No disadvantages | 1 | 0 | 3 |
| Adverse reactions | 3 | 5 | 4 |
| High cost | 2 | 3 | 2 |
| Requires trained and skilled staff | 1 | 1 | 3 |
| Requires equipment | 0 | 1 | 2 |
| Requires time to administer | 2 | 2 | 0 |
| Requires monitoring IV infusion | 1 | 3 | 0 |
| Availability of emergency drugs for adverse events | 0 | 1 | 0 |
| Complexity |  |  |  |
| Challenges in providing FCM IV iron to postpartum women with moderate anemia |  |  |  |
| No challenges | 3 | 1 | 0 |
| Length of time to administer IV iron | 2 | 1 | 3 |
| Number of steps to follow | 2 | 4 | 1 |
| Length of time to train workers to administer IV iron | 0 | 1 | 1 |
| Not enough trained or skilled workers to administer IV iron | 1 | 3 | 4 |
| Woman needs to be monitored constantly | 1 | 3 | 3 |
| Ability to manage adverse events during or after infusion | 2 | 3 | 3 |
| Availability of emergency drugs for adverse events | 1 | 2 | 0 |
| Woman or family needs convincing | 0 | 0 | 3 |
| Providing FCM IV iron is more difficult for me as a health worker than giving oral iron to postpartum women (Health workers only) | **N=3** | **N=3** | **N=4** |
| Strongly disagree | 2 | 0 | 2 |
| Disagree | 0 | 1 | 0 |
| Undecided | 0 | 0 | 1 |
| Agree | 0 | 2 | 1 |
| Strongly agree | 1 | 0 | 0 |
| How easy or difficult is it to administer FCM IV iron compared with providing other services |  |  |  |
| Very difficult | 0 | 0 | 0 |
| Difficult | 0 | 1 | 0 |
| Neutral | 0 | 2 | 1 |
| Easy | 5 | 2 | 4 |
| Very easy | 0 | 0 | 1 |
| **Outer setting** | **N=5** | **N=5** | **N=6** |
| Patient needs and resources |  |  |  |
| What are the needs and preferences of postpartum women related to anemia treatment |  |  |  |
| They prefer IV iron | 1 | 2 | 2 |
| They prefer oral iron | 4 | 2 | 1 |
| They prefer both IV iron and oral iron | 0 | 0 | 0 |
| They prefer nutrition counseling | 0 | 0 | 3 |
| Other | 0 | 1 | 0 |
| Before the PRIORITY trial, how aware were you/hospital staff of the needs and preferences of postpartum women with moderate anemia |  |  |  |
| Not aware at all | 1 | 0 | 0 |
| Slightly aware | 0 | 1 | 0 |
| Somewhat aware | 1 | 0 | 0 |
| Moderately aware | 2 | 3 | 3 |
| Extremely aware | 1 | 1 | 3 |
| The needs and preferences of postpartum women served by this hospital were considered before implementing the PRIORITY trial | 4 | 5 | 6 |
| How well do you think the FCM IV iron used in this trial meets the needs of postpartum women with moderate anemia |  |  |  |
| Not well at all | 0 | 0 | 0 |
| Slightly well | 0 | 1 | 0 |
| Somewhat well | 1 | 0 | 0 |
| Moderately well | 1 | 1 | 1 |
| Extremely well | 3 | 3 | 5 |
| Barriers postpartum women face to receiving IV iron |  |  |  |
| No barriers | 2 | 2 | 0 |
| Fear of injection | 3 | 3 | 2 |
| Cost | 1 | 0 | 2 |
| Think it is ineffective | 0 | 0 | 2 |
| Concern of side effects to self | 3 | 2 | 4 |
| Concern of side effects to child | 3 | 1 | 2 |
| Other | 0 | 2 | 4 |
| External policies (Administrators only) | **N=2** | **N=2** | **N=2** |
| What factors influenced the decision to implement the PRIORITY trial in this hospital |  |  |  |
| Nothing influenced the decision | 0 | 1 | 0 |
| Hospital was approached to participate in the trial | 2 | 0 | 2 |
| Local policies | 2 | 1 | 0 |
| State policies | 0 | 1 | 2 |
| National policies | 0 | 0 | 2 |
| Hospital clinical performance measures | 1 | 0 | 0 |
| Financial incentive | 0 | 0 | 0 |
| Other | 0 | 1 | 2 |
| **Inner setting** | **N=5** | **N=5** | **N=6** |
| Relative priority |  |  |  |
| How much of a priority is FCM IV iron for postpartum women compared with other health services |  |  |  |
| Not a priority | 0 | 0 | 0 |
| Low priority | 0 | 0 | 0 |
| Medium priority | 0 | 1 | 1 |
| High priority | 3 | 4 | 4 |
| Essential | 2 | 0 | 1 |
| Readiness for implementation |  |  |  |
| How open are you/hospital staff to providing FCM IV iron to postpartum women after the PRIORITY trial ends |  |  |  |
| Not at all receptive | 0 | 0 | 0 |
| Slightly receptive | 0 | 0 | 0 |
| Somewhat receptive | 0 | 0 | 0 |
| Moderately receptive | 0 | 3 | 0 |
| Extremely receptive | 5 | 2 | 6 |
| Available resources |  |  |  |
| The hospital has sufficient resources to implement and administer FCM IV iron after the trial ends | 4 | 1 | 2 |
| After the PRIORITY trial ends, how much of a barrier will procuring necessary resources be to providing FCM IV iron to women postpartum |  |  |  |
| Not a barrier | 0 | 2 | 0 |
| Somewhat of a barrier | 2 | 0 | 3 |
| Moderate barrier | 3 | 0 | 3 |
| Extreme barrier | 0 | 3 | 0 |
| **Individual** | **N=5** | **N=5** | **N=6** |
| Knowledge and beliefs about intervention |  |  |  |
| How knowledgeable are you about FCM IV iron implementation |  |  |  |
| Not at all knowledgeable | 0 | 0 | 0 |
| Slightly knowledgeable | 0 | 1 | 0 |
| Somewhat knowledgeable | 0 | 0 | 0 |
| Moderately knowledgeable | 2 | 2 | 3 |
| Extremely knowledgeable | 3 | 2 | 3 |
| Self-efficacy |  |  |  |
| How confident are you/hospital staff can successfully administer FCM IV iron to postpartum women |  |  |  |
| Not confident at all | 0 | 0 | 0 |
| Slightly confident | 0 | 0 | 0 |
| Somewhat confident | 0 | 1 | 0 |
| Fairly confident | 1 | 1 | 0 |
| Completely confident | 4 | 3 | 6 |
| **Proctor’s Implementation Outcomes** | **n** | **n** | **n** |
| **Acceptability** | **N=5** | **N=5** | **N=6** |
| Providing FCM IV iron to postpartum women is acceptable to me |  |  |  |
| Completely disagree | 0 | 0 | 0 |
| Disagree | 0 | 0 | 0 |
| Neither agree nor disagree | 0 | 0 | 0 |
| Agree | 0 | 3 | 1 |
| Completely agree | 5 | 2 | 5 |
| I have no objection to providing FCM IV iron to postpartum women |  |  |  |
| Completely disagree | 0 | 0 | 0 |
| Disagree | 0 | 0 | 0 |
| Neither agree nor disagree | 0 | 0 | 0 |
| Agree | 0 | 1 | 2 |
| Completely agree | 5 | 4 | 4 |
| **Feasibility** | **N=5** | **N=5** | **N=6** |
| Providing FCM IV iron to postpartum women seems implementable |  |  |  |
| Completely disagree | 0 | 0 | 0 |
| Disagree | 0 | 1 | 0 |
| Neither agree nor disagree | 0 | 0 | 0 |
| Agree | 4 | 3 | 4 |
| Completely agree | 1 | 1 | 4 |
| Providing FCM IV iron to postpartum women seems easy |  |  |  |
| Completely disagree | 0 | 0 | 0 |
| Disagree | 0 | 0 | 0 |
| Neither agree nor disagree | 0 | 1 | 1 |
| Agree | 2 | 1 | 1 |
| Completely agree | 1 | 1 | 2 |
