# Supplementary material for: Using implementation research frameworks to identify facilitators and barriers to implementation of ferric carboxymaltose intravenous iron among moderately anemic postpartum women participating in a trial in India
Source: PLoS One. 2026 Oct 21;21(10):e0359787. doi: 10.1371/journal.pone.0359787 (PMC13686276; doi:10.1371/journal.pone.0359787)
Supplement: Supplementary file 5 [file pone.0359787.s005.docx]

**S5 Table.** Summary of Quantitative and Qualitative Results for CFIR Constructs and Proctor’s Implementation Outcomes

|  | **Quantitative Findings** | **Qualitative Findings** | **Convergence/**  **Divergence** |
| --- | --- | --- | --- |
| **CFIR domain/construct** |  |  |  |
| **Intervention characteristics** |  |  |  |
| Evidence strength and quality | PRIORITY trial training and practice guidelines were main sources of information about FCM IV iron. This information made health staff more confident providing IV iron. | Health staff described learning about FCM IV iron from training and other sources and how that increased their confidence. | Convergence |
| Relative advantage | Most health staff said IV iron is better than oral iron because patient compliance is assured and there is a single dose. | Health staff explained that a single dose at the hospital ensures compliance. | Convergence |
| Complexity | Health staff said there were not enough trained workers to administer IV iron, manage potential adverse events, or monitor women during infusion. They said it was easy to administer IV iron compared to other services, but about half said it was more difficult than giving oral iron. | Health staff said that monitoring women for half an hour is difficult because of their workload. Challenges related to skills could be addressed through regular training. | Convergence |
| **Outer setting** |  |  |  |
| Patient needs and resources | Most health staff said they were aware of women’s needs and preferences, and that use of FCM IV iron meets women’s needs. Health staff identified women’s concern of side effects and fear of injection as possible barriers to receiving IV iron. | Health staff talked about the importance of counseling women about FCM IV iron and explaining that side effects are rare to address their worries. | Convergence |
| External policies (administrators only) | Administrators said that the hospital was approached to participate in the intervention. | Administrators mentioned the importance of implementing this intervention with full government support. | Convergence |
| **Inner setting** |  |  |  |
| Relative priority | Most health staff said FCM IV iron is a priority compared to other health services. | Health staff talked about the importance of effective postpartum treatment for anemia because it affects both the mother and baby. | Convergence |
| Readiness for implementation | Health staff are receptive to providing FCM IV iron after the trial ends. | The hospitals in the trial already have designated space and equipment for adverse events. They need the government to provide the FCM IV iron. | Convergence |
| Available resources | More than half of health staff said that procuring resources for FCM IV iron would be a moderate or extreme barrier. | Hospitals need access to FCM IV iron. They have the other necessary supplies and equipment. | Convergence |
| **Individual** |  |  |  |
| Knowledge and beliefs about intervention | Most health staff felt knowledgeable about FCM IV iron implementation. | Women who received FCM IV iron believed that it would reduce their anemia and make them feel more energetic. | Convergence |
| Self-efficacy | Most health staff were completely confident about administering FCM IV iron. | Health staff said that their experience giving FCM IV iron during the trial increased their confidence in implementing it. | Convergence |
| **Implementation process** |  |  |  |
| Formally appointed internal implementation leaders | There were no appointed implementation leaders. | Health workers said that the chief medical officer, doctors, and nurses need to be onboard for FCM IV iron implementation. | Convergence |
| **Proctor’s outcomes** |  |  |  |
| **Acceptability** | All health staff said providing FCM IV iron is acceptable to them. 90% of women said FCM IV iron met their expectations and >85% said they would get it again if needed. | Women generally found FCM IV iron acceptable. Health staff said that they had to explain the benefits of FCM to some women before they agreed to receive it. | Convergence |
| **Feasibility** | Most health staff said FCM IV iron is implementable and providing it seems easy. >90% of women said getting IV iron was easy and >98% faced no barriers to receiving it. | Health staff responses related to feasibility conflict with issues related to complexity. Women said that getting one infusion was easier than taking oral iron for six months. | Divergence between feasibility and complexity |
| **Perceived cost** | ~75% of women said they are willing to pay for FCM IV iron in the future, but the amount they can pay is low. | Women mentioned being able to pay a small amount for FCM or asked for part of the cost to be paid by the government. | Convergence |
| **Fidelity** | Observed fidelity of implementation was high. | - | - |
